# SIDERITE: Unveiling hidden siderophore diversity in the chemical space through digital exploration

**DOI:** 10.1002/imt2.192

**Published:** 2024-04-05

**Authors:** Ruolin He, Shaohua Gu, Jiazheng Xu, Xuejian Li, Haoran Chen, Zhengying Shao, Fanhao Wang, Jiqi Shao, Wen‐Bing Yin, Long Qian, Zhong Wei, Zhiyuan Li

**Affiliations:** ^1^ Center for Quantitative Biology, Academy for Advanced Interdisciplinary Studies Peking University Beijing China; ^2^ Peking‐Tsinghua Center for Life Sciences, Academy for Advanced Interdisciplinary Studies Peking University Beijing China; ^3^ Jiangsu Provincial Key Lab for Solid Organic Waste Utilization, Key Lab of Organic‐Based Fertilizers of China, Jiangsu Collaborative Innovation Center for Solid Organic Wastes, Educational Ministry Engineering Center of Resource‐Saving Fertilizers Nanjing Agricultural University Nanjing China; ^4^ Beyond Flux Technology Co., Ltd. Beijing China; ^5^ State Key Laboratory of Mycology, Institute of Microbiology Chinese Academy of Sciences Beijing China; ^6^ Savaid Medical School University of Chinese Academy of Sciences Beijing China

## Abstract

In this work, we introduced a siderophore information database (SIDERTE), a digitized siderophore information database containing 649 unique structures. Leveraging this digitalized data set, we gained a systematic overview of siderophores by their clustering patterns in the chemical space. Building upon this, we developed a functional group‐based method for predicting new iron‐binding molecules with experimental validation. Expanding our approach to the collection of open natural products (COCONUT) database, we predicted a staggering 3199 siderophore candidates, showcasing remarkable structure diversity that is largely unexplored. Our study provides a valuable resource for accelerating the discovery of novel iron‐binding molecules and advancing our understanding of siderophores.

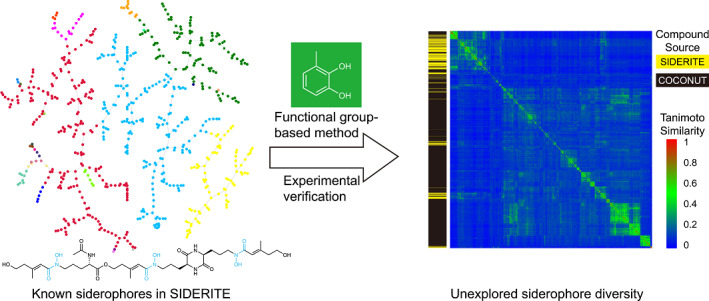

Siderophore is a diverse family of secondary metabolites that exhibit high affinities for binding and chelating iron, one of the essential elements for cellular processes, including replication and respiration [1, 2]. The significance of siderophores lies in their vital role in ensuring microbial survival and growth. Pathways associated with siderophore synthesis and uptake are widely present in microorganisms [3], constituting complex ecological games [4]. As a special type of natural product, siderophores exhibit notable antibacterial and antifungal activities, making them promising candidates for the development of novel therapeutics [5]. Despite their importance, our current understanding of siderophores is still limited due to their high diversity.

Thanks to the efforts of countless researchers over the past few decades, significant progress has been made in systematically analyzing siderophores. In 2010, Robert C. Hider and Xiaole Kong provided a valuable resource of siderophores in a seminal review, which included structural features of 294 siderophores in the appendix [6]. According to a review in 2014, over 500 different types of siderophores have been identified, with 270 having been structurally characterized [7]. The only siderophore database (http://bertrandsamuel.free.fr/siderophore_base/index.php), which contained 262 siderophores, was last updated in 2013 and is no longer maintained. Moreover, new siderophore molecules and even new siderophore functional group types [8] are constantly being discovered in various microorganisms. Information regarding these siderophores is currently dispersed across various publications and needs to be systematically recorded.

Another significant challenge in achieving a systematic overview of siderophores is the lack of digitalization, which hinders computational investigations. In the field of natural products, large digitized databases such as collections of open natural products (COCONUT) [9] record their molecules in Simplified Molecular Input Line Entry System (SMILES) format. SMILES is the commonly used format for storing and analyzing chemical molecules, which translates a chemical structure into a string of symbols that are easily readable by computer software [10]. This format enables large‐scale computational investigations, such as machine learning [10]. However, there is no systematically curated digital data set about siderophores. Digitalized natural product databases do not offer publicly accessible and searchable instances of “siderophore” and only contain a fraction of currently known siderophores.

Taken together, establishing a comprehensive siderophore database is crucial for gaining a deeper understanding of siderophore synthesis, function, and application. To fulfill this need, we have developed the Siderophore Information Database (SIDERTIE), a user‐friendly platform that includes 649 unique structures in SMILES format, covering all known siderophores up to May 2023. Leveraging SIDERTIE's digitalization capabilities, we presented the most comprehensive statistics of siderophores to date, covering biosynthetic pathways, source of producers, and several chemical characterizations. The dispersed distribution of known siderophores within the chemical landscape of natural products hints at the vast, largely uncharted territory of undiscovered diversity. Building upon this quantitative overview, we proposed a functional group‐based method to batch discover new siderophores, with experimental validation.

## RESULTS AND DISCUSSION

### Overview of SIDERITE

The Siderophore Information Database (SIDERITE, http://siderite.bdainformatics.org) contains 872 records covering all known siderophores up to May 2023. In addition to siderophore records from previous databases and reviews [6, 11, 12, 13, 14], 224 records were curated from single research articles for the first time (Table S1). In addition to the expanded collection, SIDERITE records the siderophore structures in SMILES format. Notably, in comparison to other siderophore collections, SIDERITE boasts the largest collection of siderophores and stands out for being freely accessible and digitized (Table S2). Digitizing siderophores enables computational analysis, particularly in unifying siderophores based on their chemical structures. By comparing the canonical SMILES of siderophores, we identified 649 unique siderophore structures out of the 872 total records (Table S3). During this process, we observed that many siderophores share identical structures but have different names, such as bacillibactin and corynebactin. This observation indicates that the same siderophores were discovered in different species or by different research groups [15, 16]. Therefore, for each unique siderophore structure, we merged corresponding records and designated one of their names as the official “Siderophore name” while recording the other names as “Siderophore other name.” Digitizing siderophores also enables computational analysis of statistics (Figure S1).

### Clustering of siderophores by structural similarities

**Figure 1:**
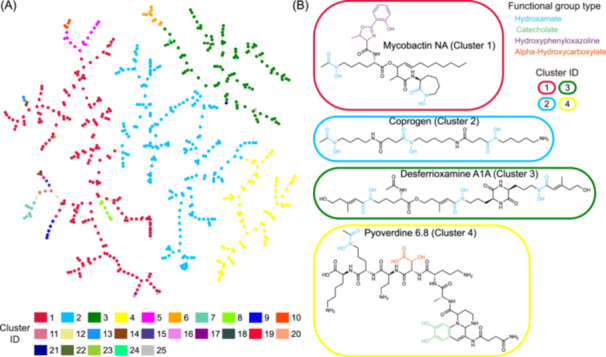
The visualization of 25 siderophore clusters in the siderophore information database (SIDERITE). (A) A network of siderophores connected by chemical similarities. Each node in the network corresponds to a siderophore molecule, and the nodes are linked to their most similar neighbors, forming a minimum‐spanning tree (described in the method section). Nodes are colored by their cluster IDs which are indexes of 25 siderophore clusters (described in the main text). (B) For the four largest clusters, example structures are provided, and the functional groups of the siderophores are colored according to their types. Siderophores are circled by rounded rectangles to show their cluster IDs (same color codes as (B)).

Siderophores have been known to exhibit remarkable structural diversity [6] (Figure S2). Converting siderophores into SMILES format enables us to quantify their chemical similarity more effectively, both within the SIDERITE database and between other natural products. To systematically assess the structural diversity of siderophores, we first locate all 649 SIDERITE structures in the vast chemical space of the COCONUT database by merging all molecules in these two databases, which encompass over 4 × 10^5^ natural products. By TMAP visualization of chemical similarity (Figure S3, described in the method section), we observed that the 649 siderophores could be grouped into 25 distinct clusters, which were separated from each other by natural products from the COCONUT. The clustering result shows that siderophores have unevenly distributed structural diversity (Figure 1 and Table S3). Most of these clusters (16 out of 25) only contain a few members (<5), while the largest four clusters account for 89.37% of the siderophore structures in total. We sorted the cluster indexes by their member counts in descending order; for instance, cluster 1 contains the most siderophore structures.

Within each cluster, there are common features of functional groups or biosynthetic types. Cluster 1 (201, 30.97%) includes siderophores are synthesized by both nonribosomal peptide synthetase (NRPS) and NRPS‐independent siderophore (NIS) with phenyl ring structures (e.g., catecholate and phenolate) in the functional groups. Cluster 2 (197, 30.35%) only includes siderophores produced by NRPS. Most siderophores in cluster 2 contain hydroxamate (92.39%, 182/197) and alpha‐hydroxycarboxylate (37.06%, 73/197). Cluster 3 (103, 15.87%) consists entirely of NIS siderophores. Like cluster 2, most of them contain hydroxamate (90.29%, 93/103), and many also contain alpha‐hydroxycarboxylate (33.01%, 34/103). Cluster 4 (79, 12.17%) is NRPS siderophores with chromophores, such as pyoverdines (93.67%, 74/79). Other small clusters are all located on the edge of four large clusters (Figure 1A). They consist of similar functional group composition (Figures S4–S16), which indicates the possibility of unusual siderophores evolving from common siderophores.

### Naming siderophore by clusters and groups

Clusters only provide an initial classification relative to other natural product molecules. Further, we defined groups within each of the 25 clusters by their structure similarity coefficient and obtained 102 groups in total. Each group is named by its cluster ID *x* and its group ID *y* within this cluster. Accordingly, each siderophore is assigned a unique ID *x.y.z*, where *z* stands for the *z*th record within the group. In the future, newly discovered siderophores will also be assigned a unique ID when being incorporated into the SIDERITE database.

For siderophores with unknown biosynthetic types, their biosynthetic types could be inferred by other members with known biosynthetic types in the same group because almost all members in the same group have the same biosynthetic types (except for 1.5 and 1.10 groups). It is useful for mining their biosynthetic genes in the genome after discovering new siderophores, which would accelerate siderophore research from structures to genes.

### Discovering potential siderophores in silico by the functional group‐based method

**Figure 2:**
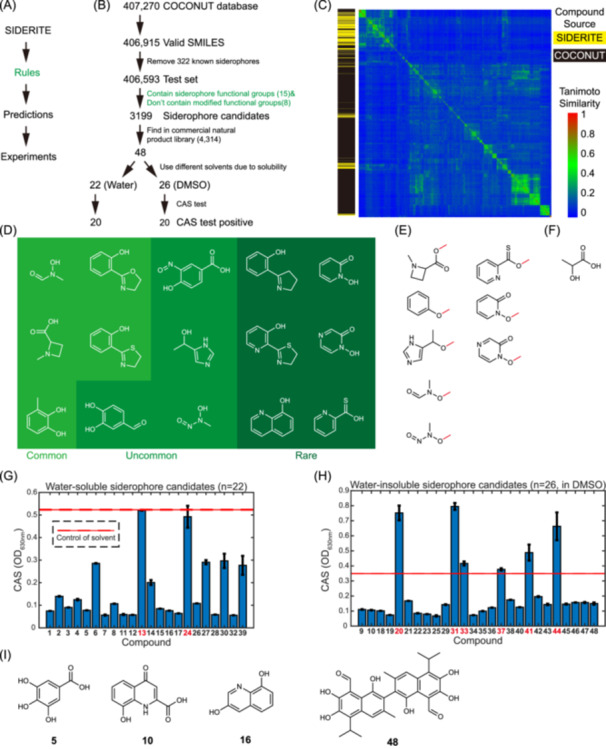
The rule‐based siderophore discovery approach and the result of chrome azurol S (CAS) test experiments. (A) Principle of rule‐based siderophore discovery approach. The rules are summarized from known siderophores based on the SIDERITE database. The predictions were then tested by experiments. (B) Pipeline of the functional group‐based siderophore discovery. Molecules containing at least one of 15 functional groups in (D) and not any of the eight modified functional groups in (E) are selected as siderophore candidates. (C) The structural diversity of new potential siderophores in the collection of open natural products (COCONUT) database. In all, 3199 molecules with potential iron‐binding activities and 649 known siderophores were clustered with Tanimoto similarity. The source of molecules (COCONUT or SIDERITE) is shown in the left bar by yellow and black colors, respectively. (D) The structures of siderophore functional groups in the rules in (B). The rarity of functional groups is noted by the different background colors. (E) The structures of modified functional groups in the rules in (B). Modifications that cause functional groups to lose iron‐chelating abilities are marked in red. (F) The structure of common functional group alpha‐hydroxycarboxylate. (G) The CAS result of the 25 water‐soluble siderophore candidates. The solvent is water. The mean and ± one standard deviation of optical density (OD, 630 nm) are shown in the bar graph. The OD (630 nm) of solvent is marked as a red line (mean value) and a red dash line (one standard deviation). The compounds with negative results in the CAS test are marked in red. (H) The CAS result of 30 water‐insoluble siderophore candidates. The solvent is dimethyl sulfoxide (DMSO). Other legends are the same as (F). (I) The representative examples of compounds with iron‐binding activities.

The clustering analysis of known siderophores has unveiled the presence of common functional groups among these compounds. Drawing inspiration from this observation, we have introduced an innovative rule‐based approach aimed at the discovery of novel siderophores by their chemical structures (Figure 2A–C). In this approach, we first distilled 15 common functional groups derived from the characteristics of known siderophores. Any molecule containing at least one of these 15 functional groups is identified as a potential siderophore (Figure 2D and Table S4). Then, we exclude candidates containing any of the eight modified siderophore functional groups incapable of forming coordination bonds to chelate iron (Figure 2E and Table S5). Besides, while the alpha‐hydroxycarboxylate functional group is prevalent within siderophores (Figure 2F), we have excluded it from our rule set due to its ubiquity in nonsiderophore molecules.

To verify this method, we applied our functional group‐based method to the large chemical database COCONUT, excluding 322 known siderophores from consideration. Remarkably, this analysis led to the identification of 3199 molecules exhibiting potential iron‐binding activities from a database containing over 0.4 million natural products (Tables S2 and S6). By the Tanimoto similarity clustering (Figure 2C), a large proportion of these potentially iron‐binding molecules is scattered in the chemical space, not close to any of the known siderophores. Specifically, only 284 out of the 3199 molecules exhibit significant structural similarity (with a maximum Tanimoto similarity of >60%) to the known 649 siderophores cataloged in SIDERITE. The remaining 2915 molecules are strong candidates for novel siderophores with relatively unexplored chemical structures. This analysis underscores the notion that the structural diversity of siderophores remains largely concealed, inviting further in‐depth exploration and investigation.

### Verifying potential siderophores by chrome azurol S (CAS) assay

Subsequently, we searched for purchasable molecules out of the 3199 candidates for experimental verification. 48 molecules (Table S7 and Figure 2B) are available in the commercial natural product library (the Natural Product Library for high throughput screening, catalog number L6000, TargetMol, June 2023). Among these molecules, 22 are soluble in water, while the remaining 26 have poor solubility (Table S7). To address this, we dissolved the poorly soluble molecules by dimethyl sulfoxide instead of water. Subsequently, solutions of these 48 molecules were tested by the CAS assay, a universal colorimetric method that detects iron‐binding molecules [17].

The high positive rate from the CAS assay supports the effectiveness of our functional group‐based method. Among the tested molecules, 20 out of 22 (90.9%) water‐soluble compounds and 20 out of 26 (76.9%) water‐insoluble compounds exhibited iron‐binding activity, as evidenced by a noticeable change in the color of the CAS dye (Figure 2G,H and Table S8). Actually, most molecules with negative CAS results (compounds **20, 24, 31, 33, 37, 41**, and **44**) exhibited unusual color patterns, which hinders accurate assessment. For instance, their original solutions were significantly dark in color or reacted with CAS reagents and formed precipitates or turbidity. Actually, compounds **24, 37, 41**, and **44** did induce color change in the CAS assay solution, but their precipitation interfered with the optical density measurement. Taken together, only one (compound **13**) out of the 48 molecules was confirmed to lack iron‐chelating ability.

## CONCLUSION

In our work, we introduced the most comprehensive Siderophore Information Database (SIDERTE, https://siderite.bdainformatics.org/), the first digitized siderophore repository with 649 unique structures in the SMILES format. This digitized repository empowers researchers to transcend the limitations of manual approaches, paving the way for data‐driven discoveries in the siderophore field. On the basis of these digitized structures, a computational method was developed for discovering novel iron‐binding molecules with high accuracy and found remarkable structural diversity largely uncharted in the realm of siderophore research. SIDERTE provides a repository for novel siderophore discoveries. We provide tutorial materials and feedback channels in the database or the GitHub page (see Supporting Information Material for details) and are committed to maintaining the SIDERITE database continually and updating it based on the feedback received from our users.

## AUTHOR CONTRIBUTIONS

Ruolin He and Shaohua Gu collected the data and drafted the manuscript. Ruolin He performed the majority of computational analysis in this research. Shaohua Gu and Zhong Wei designed experiments. Ruolin He, Fanhao Wang, and Jiqi Shao proposed the functional group‐based method. Jiazheng Xu and Zhengying Shao were responsible for the execution and analysis of the CAS experiments. Xuejian Li, Haoran Chen, and Long Qian contributed to constructing the website SIDERITE. Wen‐Bing Yin offered insightful comments. Wen‐Bing Yin, Zhong Wei, and Zhiyuan Li revised the manuscript. Zhong Wei and Zhiyuan Li oversaw the project. Zhiyuan Li conceptualized the project. All authors have read the final manuscript and approved it for publication.

## CONFLICT OF INTEREST STATEMENT

The authors declare no conflict of interest.

## ETHICS STATEMENT

No animals or humans were involved in this study.

## Supporting information


**Figure S1**: The statistics of 649 unique siderophores in SIDERITE.
**Figure S2**: Known siderophore functional groups (ligands).
**Figure S3**: Displaying 25 clusters of 649 siderophores in the COCONUT database by TMAP.
**Figure S4**: Visualization of 649 siderophores with functional group hydroxamate number by TAMP.
**Figure S5**: Visualization of 649 siderophores with functional group catecholate number by TAMP.
**Figure S6**: Visualization of 649 siderophores with functional group phenolate number by TAMP.
**Figure S7**: Visualization of 649 siderophores with functional group carboxylate number by TAMP.
**Figure S8**: Visualization of 649 siderophores with functional group carboxylate in citrate number by TAMP.
**Figure S9**: Visualization of 649 siderophores with functional group alpha‐hydroxycarboxylate number by TAMP.
**Figure S10**: Visualization of 649 siderophores with functional group hydroxyphenyloxazoline number by TAMP.
**Figure S11**: Visualization of 649 siderophores with functional group hydroxyphenylthiazoline number by TAMP.
**Figure S12**: Visualization of 649 siderophores with functional group alpha‐aminocarboxylate number by TAMP.
**Figure S13**: Visualization of 649 siderophores with functional group alpha‐hydroxyimidazole number by TAMP.
**Figure S14**: Visualization of 649 siderophores with functional group alpha‐hydroxycarboxylate in citrate number by TAMP.
**Figure S15**: Visualization of 649 siderophores with functional group diazeniumdiolate number by TAMP.
**Figure S16**: Visualization of 649 siderophores with functional group 2‐nitrosophenol number by TAMP.
**Figure S17**: The predicted properties of 649 siderophores.
**Figure S18**: The distribution of C/N and C/O ratios in the different biosynthetic types and clusters.
**Figure S19**: The distribution of nitrogen atom and oxygen atom numbers in the different biosynthetic types and clusters.
**Figure S20**: The SIDERITE database usage and interface.

**Table S1**: 872 siderophore information records.
**Table S2**: Comparison of databases related with siderophores.
**Table S3**: Detailed information on 649 siderophores with unique structures.
**Table S4**: 15 functional group structures used in potential siderophore search.
**Table S5**: 8 modified siderophore functional groups in the negative control.
**Table S6**: Tanimoto similarity matrix between 649 siderophores in SIDERITE and 3199 molecules with potential iron‐binding activities in COCONUT database.
**Table S7**: Detailed information on 48 molecules with potential iron‐binding activities in the CAS assay test.
**Table S8**: The raw OD630 values of 48 molecules with potential iron‐binding activities in the CAS assay test.

## Data Availability

The data underlying this article are available in Zenodo at https://zenodo.org/doi/10.5281/zenodo.10369626. The codes underlying this article are available on GitHub at https://github.com/RuolinHe/SIDERITE. The database is available at http://siderite.bdainformatics.org. Supporting Information Materials (methods, figures, tables, scripts, graphical abstract, slides, videos, Chinese translated version, and updated materials) may be found in the online DOI or iMeta Science http://www.imeta.science/.
