# Supplementary material for: SIDERITE: Unveiling hidden siderophore diversity in the chemical space through digital exploration
Source: Imeta. 2024 Apr 5;3(2):e192. doi: 10.1002/imt2.192 (PMC11170966; doi:10.1002/imt2.192)
Supplement: Supplementary file 1 — Figure S1: The statistics of 649 unique siderophores in SIDERITE. Figure S2: Known siderophore functional groups (ligands). Figure S3: Displaying 25 clusters of 649 siderophores in the COCONUT database by TMAP. Figure S4: Visualization of 649 siderophores with functional group hydroxamate number by TAMP. Figure S5: Visualization of 649 siderophores with functional group catecholate number by TAMP. Figure S6: Visualization of 649 siderophores with functional group phenolate number by TAMP. Figure S7: Visualization of 649 siderophores with functional group carboxylate number by TAMP. Figure S8: Visualization of 649 siderophores with functional group carboxylate in citrate number by TAMP. Figure S9: Visualization of 649 siderophores with functional group alpha‐hydroxycarboxylate number by TAMP. Figure S10: Visualization of 649 siderophores with functional group hydroxyphenyloxazoline number by TAMP. Figure S11: Visualization of 649 siderophores with functional group hydroxyphenylthiazoline number by TAMP. Figure S12: Visualization of 649 siderophores with functional group alpha‐aminocarboxylate number by TAMP. Figure S13: Visualization of 649 siderophores with functional group alpha‐hydroxyimidazole number by TAMP. Figure S14: Visualization of 649 siderophores with functional group alpha‐hydroxycarboxylate in citrate number by TAMP. Figure S15: Visualization of 649 siderophores with functional group diazeniumdiolate number by TAMP. Figure S16: Visualization of 649 siderophores with functional group 2‐nitrosophenol number by TAMP. Figure S17: The predicted properties of 649 siderophores. Figure S18: The distribution of C/N and C/O ratios in the different biosynthetic types and clusters. Figure S19: The distribution of nitrogen atom and oxygen atom numbers in the different biosynthetic types and clusters. Figure S20: The SIDERITE database usage and interface. [file IMT2-3-e192-s001.docx]

# Supporting information to

# SIDERITE: Unveiling Hidden Siderophore Diversity in the Chemical Space Through Digital Exploration

**Running title**: Siderophore information database and application

Ruolin He^1#^, Shaohua Gu^1,2#^, Jiazheng Xu^3^, Xuejian Li^4^, Haoran Chen^4^, Zhengying Shao^3^, Fanhao Wang^1^, Jiqi Shao^1^, Wen-Bing Yin^5,6^, Long Qian^1^*, Zhong Wei^3^*, Zhiyuan Li^1,2^*

^1^Center for Quantitative Biology, Academy for Advanced Interdisciplinary Studies, Peking University, Beijing 100871, China

^2^Peking-Tsinghua Center for Life Sciences, Academy for Advanced Interdisciplinary Studies, Peking University, Beijing 100871, China

^3^Jiangsu Provincial Key Lab for Organic Solid Waste Utilization, Jiangsu Collaborative Innovation Center for Solid Organic Waste Resource Utilization, National Engineering Research Center for Organic-based Fertilizers, Nanjing Agricultural University, Nanjing 210095, China

^4^Beyond Flux Technology Co., Ltd., Beijing 100193, China

^5^State Key Laboratory of Mycology, Institute of Microbiology, Chinese Academy of Sciences, Beijing 100101, China

^6^Savaid Medical School, University of Chinese Academy of Sciences, Beijing 100049, China

^#^These authors contributed equally: Ruolin He, Shaohua Gu

*Correspondence: [zhiyuanli@pku.edu.cn](mailto:zhiyuanli@pku.edu.cn) (Zhiyuan Li), [weizhong@njau.edu.cn](mailto:weizhong@njau.edu.cn) (Zhong Wei), [long.qian@pku.edu.cn](mailto:long.qian@pku.edu.cn) (Long Qian)

### METHODS

### Siderophore information resource

A total of 872 siderophore information records were collected from various sources, including databases, reviews, and research articles. Among these, 355 records were obtained from Samuel Bertrand's Siderophore Base, while the remaining records were sourced from other databases, reviews, and research articles (Table S1). Specifically, we obtained 160 new records from the appendix of Robert C. Hider and Xiaole Kong's review, 37 new records from other reviews, 95 new records from the Dictionary of Natural Products (DNP) database (<https://dnp.chemnetbase.com/>), and 224 new records from research articles by searching with keywords such as "new siderophore", "novel siderophore", and "siderophore discovery". It is important to note that one new record which is a pyoverdine (a type of siderophore) was obtained from the LOTUS database with the keywords “pyoverdine”. This was because this database only allowed searching siderophores by their specific names such as “pyoverdine”, rather than by the general biological function such as “siderophore”.

Each siderophore information record in our dataset comprises various details, including the siderophore name, the synonym name (if any), the type and number of functional groups, the producing species, the biosynthetic type, and the structure (Table S1).

### Biosynthetic type annotation

Information on 721 siderophore biosynthetic types is available in our database, which we documented based on references. For the remaining 151 siderophores with unknown biosynthetic types, we inferred their biosynthetic types by features of different types. The biosynthetic type of the siderophore would be annotated as “Putative NIS”, if it contains monomers that only exist in the NIS siderophore such as citrate, diamine (e.g. 1,3-Diaminopropane, putrescine and cadaverine) or diamine derivatives (e.g. N-hydroxy cadaverine). The biosynthetic type of the siderophore would be annotated as “Putative NRPS” if the monomers in this siderophore all are amino acids.

### SMILES conversion from siderophores

The Simplified Molecular-Input Line-Entry System (SMILES) is a notation to describe the chemical structure of molecules using a string format which is particularly useful for subsequent processing by computer. However, in our siderophore information resource, the SMILES format of siderophore structures and their annotations were only available in the DNP database. In contrast, the literature and Siderophore Base only included siderophore structures in the picture format which is more intuitive for readers but poses difficulties for computational analysis. To address this limitation, we developed a customized Python script that utilized the ChemSpider API and Chemical Identifier Resolver (CIR) to convert siderophore names into the SMILES format. The SMILES format structures of 34.93% (124/355) siderophore records in the Siderophore Base were retrieved. For the remaining 748 siderophores that were not found or from other resources, we manually drew their structures and obtained the SMILES format structures by SMILES generator/checker tool (<http://www.cheminfo.org/flavor/malaria/Utilities/SMILES_generator___checker/index.html>).

To ensure consistency and facilitate comparison, we then converted all SMILES strings into canonical SMILES strings using another custom Python script. The RDKit package was utilized for this conversion process, while simultaneously obtaining the molecular formula and molecular weight of the siderophores.

### Prediction of aqueous solubility and the diffusion coefficient for siderophores based on SMILES

To predict aqueous solubility, we used a machine learning tool SolTranNet with SMILES of siderophores as input[1]. For the prediction of the diffusion coefficient, we used the SEGWE calculator developed by Evans, R. *et al*.[2] with a temperature 298.15K and water as solvent.

### Visualization of siderophore distribution in the chemical space

To compare and analyze siderophores intuitively, we visualize SIDERITE with other chemical databases by TMAP[3]. The visualization mapping of SIDERITE with other chemical databases consists of the vast chemical spaces for exploring new siderophores. The TMAP mapping algorithm tends to produce a significant distance between a molecule with and without specification of stereoisomersnon-stereoisomeric counterpart. To avoid this bias, we removed stereoisomerism information based on the canonical SMILES. The intersection of SIDERITE with other chemical databases is removed from the chemical database in the visualization.

### Clustering of siderophores

The identification of clusters of siderophores was based on their arrangement within the vast chemical space. The chemical space is composed of numerous molecules, and in our study, we employed the COCONUT database[4] which comprises approximately 407,000 small molecules. Within this space, siderophores were found to be grouped into distinct clusters, separated from other non-siderophore molecules.

To identify groups within each large siderophore cluster, we utilized a molecular structure similarity threshold of 60%. Specifically, siderophores were considered to be part of the same group if their molecular similarity was greater than 60%, as determined using the Dice coefficient (Dc)[5].

### Testing iron-binding activity by CAS test experiment

We found 3199 potential siderophores from COCONUT natural product database by our functional group-based siderophore discovery method. Among them, 48 molecules (Table S7 and Figure 2B) are available in the commercial natural product library (the Natural Product Library for high throughput screening, catalog number L6000, TargetMol, Shanghai, China, June 2023). Subsequently, we procured these small molecules (Table S7) to test iron-binding activity. The small molecules were then prepared by dissolving them in sterilized deionized water to achieve a final concentration of 1 mg/ml in an aqueous solution.

These aqueous solutions were employed to assess the iron-binding activity through the CAS test experiment[6]. Briefly, the liquid form of the CAS assay was employed, wherein 100 µl of the small molecule aqueous solution (four biological replicates for all small molecules) or sterilized deionized water as a control reference was added to 100 µl of the CAS assay solution in a 96-well plate. Following a static incubation of 2 hours at room temperature, the OD630 readings of the small molecule aqueous solution and sterilized deionized water and dimethyl sulfoxide (DMSO) controls were measured using a SpectraMax M5 plate reader at room temperature. Small molecules exhibiting siderophore iron-chelating activity would induce a color change in the CAS medium, leading to decreased OD630 measurements. Consequently, the ability of small molecules to chelate iron can be quantified using the OD630 measurements.

### RESULTS

### Statistics of siderophores in SIDERITE

#### Producer source

The 649 unique siderophores in our database can be classified by their producer sources (Figure S1A). At the kingdom level, the majority of 649 siderophores in the database are produced by bacteria (85.90%), followed by fungi (12.40%), plants (1.54%), and animals (0.15%). While most siderophores are specific to one producer source at the kingdom level, there is one exception. Triacetylfusarinine can be produced by both bacteria (*Paenibacillus triticisoli*) and fungi (*Penicillium* sp.). At the phyla level, 872 producers of 649 siderophores are spread across five major bacterial phyla (229 in Actinobacteria, 7 in Cyanobacteria, 33 in Firmicutes, 412 in Proteobacteria, and 8 in Bacteroidetes) and three major fungal phyla (124 in Ascomycota, 38 in Basidiomycota and, 8 in Mucoromycota, Table S1).

#### Biosynthetic pathway

Siderophores can also be classified by their biosynthetic pathways (Figure S1B). Previous studies have suspected that the NPRS pathway is more dominant for siderophore synthesis[7], yet no statistically concrete ratio between NRPS and NIS-derived siderophore has been established. In SIDERITE, we found that related pathways can be classified into NRPS (65.18%), NIS (21.73%), Hybrid NRPS/PKS (10.02%), Hybrid NRPS/NIS (2.77%), and PKS (0.31%). Consistent with previous studies, NRPS was indeed the most abundant biosynthetic pathway of siderophores, followed by NIS. PKS siderophores are rare, with only two cases (proferrorosamine A and tetracycline). In different kingdoms, the composition of the biosynthetic pathway differs (Table S3). In plants and animals, all siderophores are synthesized by NIS. In fungi, almost all siderophores are synthesized by NRPS (90.12%, 73/81). In bacteria, the diversity of the siderophore biosynthetic pathways is higher, with 62.90% (351/558) NRPS, 22.58% NIS (126/558), 11.29% hybrid NRPS/PKS (63/558), and 2.87% hybrid NRPS/NIS (16/558).

#### Functional group

Digitization also enables us to easily access the statistical properties of siderophores, such as functional group distribution. Siderophores chelate iron by several common functional groups (coordinating groups) [7], and a single siderophore may use multiple types of functional groups. In SIDERITE, the top five combinations of functional groups are present in 64.25% of the siderophores (Figure S1C). These top five combinations are Hydroxamate (28.35%), Alpha-Hydroxycarboxylate+Hydroxamate (12.02%), Catecholate+Hydroxamate (8.78%), Hydroxamate+Hydroxyphenyloxazoline (8.63%) and Catecholate (6.47%). Hydroxamate and Catecholate are the most common functional groups found in 69.49% and 29.74% of siderophores, respectively (Figure S1D). Most of siderophores have three bidentate groups which forming octahedral geometry with iron in coordinating number six [7]. As expected, most siderophores have a denticity number of six (Figure S1E). However, there are exceptions, such as pacifibactin, desferrioxamine T1, malleobactin D and pyoverdine 7.7, which contain four bidentate groups. It’s reported that pacifibactin coordinates iron in a 1:1 ratio, and the function of the extra coordinating group is unknown [8].

#### Molecular weight

The molecular weight (MW) is another important property of siderophores, as it influences the diffusion of siderophores [9]. Siderophores are typically small molecules, and the MW of siderophores in our database ranges from 138.12 Da (salicylic acid) to 1766.86 Da (pyoverdine IB3). Most siderophores (90.60%, 588/649) have a middle MW ranging from 300~1100 Da (Figure S1F). Of note, half of the smallest 12 siderophores (MW < 200 Da) are monomers of other siderophores, such as salicylic acid, 2,3-dihydroxybenzoic acid, and citrate. Most of the large siderophores (MW > 1200 Da) are pyoverdines (87.76%, 43/49), as pyoverdines are frequently composed of more than ten amino acids. Further, we check whether MW distribution is related to biosynthetic types. We found NRPS siderophores exhibit a wide range of MW, from 206.20 Da (Spoxazomicin D) to 1766.86 Da (pyoverdine IB3). NRPS siderophores are generally heavier (mean: 835.68 Da; median: 816.00 Da) than NIS siderophores (mean: 506.93 Da; median: 516.63 Da).

#### Aqueous solubility and the diffusion coefficient

Aqueous solubility and the diffusion coefficient also are important ecological properties of siderophores. However, most siderophores have not been measured experimentally for these two properties. Therefore, we employed SolTranNet [1] and Stokes–Einstein Gierer-Wirtz Estimation (SEGWE) [2] to predict these properties using SMILES representations (Figure S17). Out of the total 649 siderophores, 635 were predicted to be water-soluble (predicted logS>-6). Among them, 551 siderophores exhibited a “good” level of aqueous solubility (predicted logS>-4). Notably, 14 siderophores exhibit poor aqueous solubility (predicted logS≤-6) due to the presence of long-chain fatty acids, suggesting them are probably insoluble in water. The minimum and maximum predicted diffusion coefficients are 2.66×10^-10^ m^2^/s and 7.7×10^-10^ m^2^/s respectively. The majority of siderophores (611 out of 649) exhibit predicted diffusion coefficients within the range of 2.66 to 5.50×10^-10^ m^2^/s, consistent with previous research [10].

#### Molecule weight, carbon-nitrogen ratio, and carbon-oxygen ratio in the clusters

Molecule weight (MW), carbon-nitrogen ratio (C/N), and carbon-oxygen ratio (C/O) of siderophores pertain to their biosynthetic cost in various aspects: higher MW can indicate more building blocks; while producing products with lower C/N or C/O could influence growth under nitrogen-limited or hypoxic environments [11−13]. Therefore, we investigated these properties of siderophores in different clusters (Figure S18). Siderophores in cluster 4 have significantly higher MW than average, as most cluster members are pyoverdines. Regarding C/N and C/O ratios, different clusters also exhibit different properties. For example, siderophores in cluster 7 and cluster 10 have significantly higher-than-average C/N because they only contain 1~2 nitrogen atoms, and siderophores in cluster 4 have a significantly lower-than-average C/N (Figure S19).

#### Biosynthetic type in the clusters

All groups encompass siderophores derived from unique biosynthetic types, except groups 1.5 and 1.10, where siderophores are synthesized through both hybrid NRPS/PKS and NRPS pathways. The siderophores in these two groups have two biosynthetic types due to the optional modification from PKS. Also, siderophores within the same group are typically produced by species from the same kingdom. However, groups 2.1, 2.9, 3.7, and 9.1 contain producers from both bacteria and fungi, and group (11.1) is produced by bacteria and animals.

### The features of 48 molecules in the experiment

We checked the producer sources of these 48 molecules and found that, except for chlorquinaldol (compound **29**) which is synthetic [14], the other 47 are natural products derived from plant, insects, microbes, algae, or humans (Table S7). Of these 40 molecules, which we confirmed to exhibit iron-chelating capabilities (referred to as 'potential siderophores' henceforth), the majority remains largely unexplored regarding their iron-binding potential. Remarkably, only three (compounds **5**, **10**, and **48**, see Figure 2I with their seven derivatives have been previously associated with iron-binding related research, but have not yet been regarded as siderophores: 1. Gallic acid (compound **5**) has been systematically studied for complexation with iron [15] and has been commonly used in the pharmaceutical industry. In these 40 molecules, there are six gallic acid derivates (compounds **12**, **14**, **17**, **23**, **25**, and **27**) with iron-binding activity. 2. Xanthurenic acid (compound **10**) is the precursor of *Pseudomonas* siderophore quinolobactin[16]. 3. Gossypol (compound **48**) has been reported as dietary supplementation with interactions with ferrous sulfate[17], implying iron-binding activity. Gossypol derivate gossypol acetic acid (compound **47**) also shows iron-binding activity in our assay. Taken collectively, at least 30 of the molecules we identified may represent novel siderophores.

We then checked the chemical properties of these 40 potential siderophores. Out of the 40 molecules, the most frequent functional group type is catecholate (36/40). Among these, the majority (19/36) are trihydroxy, such as gallic acid (compound 5, Figure 2I). The remaining four molecules not possessing catecholate contain 8-hydroxyquinoline, which is an uncommon functional group. It is worth noting that while it is known that 8-hydroxyquinoline can chelate metal, only two siderophores (quinolobactin and thioquinolobactin) containing this functional group have been reported so far [18,19]. Therefore, our discovery of the four molecules significantly expands the list of 8-hydroxyquinoline siderophores. The four molecules are robustine (compound **9**), xanthurenic acid (compound **10**, Figure 2I), jineol (compound **16**, Figure 2I), and chlorquinaldol (compound **29**). Chlorquinaldol is a synthetic compound. Robustine is produced by plant *Zanthoxylum integrifoliolum* [20] and *Zanthoxylum avicennae* [21]. Jineol is produced by the insect *Scolopendra subspinipes* [22]. The biosynthetic pathways of robustine and jineol are currently unknown.

Regarding the functions of the 40 potential siderophores, most of them (excluding 14 molecules with unknown functions) are known to have antioxidant activities (14/40). This percentage is significantly higher than the proportion of molecules with “antioxidant” annotations in the commercial library (5.1%). The second most common function are anticancer (7/40), followed by anti-inflammatory (4/40, Table S7). The high occurrence of these pharmaceutical functions in potential siderophores motivates us to explore of the large natural product repository for additional molecules with iron-binding capabilities.

### The usage of SIDERITE database

SIDERITE is freely accessible at <http://siderite.bdainformatics.org>. The current version serves as a platform for searching and displaying known siderophore chemical structures. Users can access desired siderophores by the following three approaches (Figure S20A).

An indexing interface is provided so that users can quickly access the desired siderophores. We visualize the SIDERITE database by TMAP [3], and label multiple properties of siderophores (Figure 1A and Figure S20B, <http://siderite.bdainformatics.org/sidertmap>). In the mapping, each siderophore is represented as a distinct point that offers more detailed information when clicked. Additionally, users can jump to the siderophore page by the interactive siderophore ID in the information card (Figure S20B).

Users also can use the search function to access desired siderophores (Figure S20C, D). The platform offers fast fuzzy search functionality based on partial siderophore names, as well as precise advanced search by multiple conditions. The advanced search feature includes various conditions, including siderophore ID, siderophore name, siderophore functional group, biosynthetic type, monomer name, number of monomers, monomer coverage, theoretical denticity, molecular weight, microorganism, source (kingdom), source (phylum), and class. Numeric conditions, such as the number of monomers and molecular weight, can also be set within specified ranges.

Chemical similarity search is the third way to access the siderophore resource in SIDERITE (Figure S20E). For any query molecule, this function helps users to find structurally similar siderophores, and also predict whether this query molecule has iron-binding activity. Users can input the SMILES notation of the query molecule or draw it in the SMILES editor. If the molecule has been predicted to have potential iron-binding activity by our functional group-based method (Figure 2) and is not already present in SIDERITE, the SIDERITE platform will automatically indicate to the user that this is a potentially new siderophore. Users can choose whether or not to upload this molecule to the SIDERITE database without any obligations. Furthermore, this search will return siderophores in SIDERITE that are chemically similar to the query molecule. The chemical similarity metric supports Tanimoto, Dice, and Tversky, and the user can customize the cut-off threshold.

We provide individual pages with detailed information for each siderophore structure (Figure S20F), which provide canonical SMILES, pictures, and related references. Alternative names will be noted after the main name, separated by a forward slash. In the “Functional Group” section, the number of specific functional groups is specified within parentheses. We also provide tutorial materials and feedback channels, which can be accessed in the database or the GitHub Wiki page (<https://github.com/RuolinHe/SIDERITE/wiki/Tutorial>). Additionally, users can report bugs or pose general inquiries about SIDERITE through GitHub Issues page (<https://github.com/RuolinHe/SIDERITE/issues>) or our dedicated Google group (<https://groups.google.com/g/siderite-database>). We are committed to maintaining the SIDERITE database continually and updating it based on the feedback received from our users.

## DISCUSSION

Our work provided a statistical overview on siderophores, from the perspectives of biosynthetic type, producer source, and chemical properties. Particularly, the clustering patterns of siderophores in the chemical space hold implications for their eco-evolutionary histories [23], and the drives of their chemical diversity. Siderophores are considered public goods as they are predominantly secretive[24]. However, some species have evolved the ability to exploit siderophores without producing them, by synthesizing only the receptors for these siderophores [23,24]. To avoid this exploitation, producers can modify the structure of their siderophores [23,25,26]. This evolutionary pressure under such a Red Queen situation may generate large siderophore clusters with similar but diverse structures. For example, researchers recently found *Streptomyces venezuelae* produced the uncommon, alternative siderophore foroxymithine (cluster 2.7) to impose a more profound fitness when their main siderophore desferrioxamine (cluster 3.3) was exploited by the yeast *Saccharomyces cerevisiae* (cheater) in the co-culture [27]. Meanwhile, siderophores in clusters with few similar members may indicate other strategies, like utilizing multiple structurally distinctive siderophores to avoid intense competition. Across different kingdoms, bacteria exhibit the most diverse synthetic types and the highest number of siderophores, reflecting the intense iron competition observed among prokaryotes [8,23].

Dealing with such a high diversity, one advantages of digitalization is to facilitate the annotation of previously unnoticed siderophores. During our construction of SIDERITE, 50 siderophore records were found by exploring chemically similar natural product molecules around known siderophores on the COCONUT database mapping. Among them, 29 records are from references that do not contain "siderophore" in the title, making their detection through literature searches more challenging. In fact, half of them were initially reported as antibiotics or microbial inhibitors. Also, categorizing siderophores by the SMILES format avoid rediscovering of the same structures from different organisms yet annotating them as “new” siderophores [28−35].

Another advantage of digitalization is to provide resources for data-driven explorations, such as in bio-retrosynthesis prediction. In recent years, deep learning approaches have been employed for predicting the biosynthetic pathways of natural products [36]. In addition, high-quality labeled siderophore data can be used to train supervised learning models, leading to the design of novel artificial siderophores with enhanced biological activities.

In fact, our functional group-based method for searching iron-chelating structures serves as a preliminary prototype for data-driven discoveries. Although simple in nature, it can be seen as analogous to supervised learning, where features related to iron-binding (such as rules for including or excluding certain functional groups) are extracted from the existing SIDERITE dataset and applied to chemicals with unknown iron-chelating abilities. With the high successful rate validated by our CAS experiments, this functional group-based method is likely applicable to the vast repository of natural products. In the COCONUT database, our method predicted 3199 molecules as iron-binding compounds. Particularly, siderophore clusters only containing few members can suggest underexplored siderophore types and highlights for future research. For example, pyridoxatin has only one member in the cluster 18 of SIDERITE. Cordypyridone A and B with antimalarial activity are the nearest natural products to pyridoxatin in the chemical space of COCONUT, and both compounds were predicted as “iron-binding” by our method. However, cordypyridone A and B were not previously considered to be siderophores [37], indicating underexplored siderophore types that call for verification by subsequent experiments.

More inquiringly, most of the 3199 structures are different from known siderophores, suggesting that the true extent of siderophore diversity remains largely unexplored. Our findings also imply a higher prevalence of iron-binding molecules in plants than anticipated. These plant siderophores, known as phytosiderophores, are predominantly produced by graminaceous plants[7]. Remarkably, our data demonstrates that 92.5% (37 out of 40) of our identified iron-binding molecules originate from plants (Table S7), revealing substantial untapped potential for phytosiderophores.

Developing new methods to quickly discover new siderophores by systematically summarizing the functional group diversity and structural features of siderophores holds immense scientific and practical importance. Primarily, uncovering novel siderophores can open avenues for innovative therapeutic strategies. Iron's critical role in various diseases, including cancer and inflammation, underscores its significance [38−40]. It is known siderophores have an antioxidant role by chelating iron because iron can cause reactive oxygen species by reacting with H_2_O_2_ [41−43]. And anticancer and anti-inflammatory activities of siderophores are achieved by sequestering iron in the environment to limit the growth of cancer cells[38] and microbes [39,40]. More than half of our newly identified iron-chelating molecules have reported antioxidant, anticancer, and anti-inflammatory properties. These molecules may also be used as environmental remediation of heavy metal pollution [44,45] .

Different from previous siderophore resources, which remain limited to single studies and therefore do not provide a mechanism to perform cross-study and systematic analyses, SIDERITE provides a curated community-wide platform. All siderophores are digitized, which makes comparisons easy and thus avoids duplicate naming of the same molecule. Each siderophore is assigned a three-level serial unique id based on the siderophore structure similarity feature. The unique id assigned to each siderophore in this study also provides a standardized nomenclature for future studies. With the advent of SIDERITE, newly discovered siderophores can be easily identified and quickly known by other researchers in this field. We invite all relevant researchers to join this community and collaborate to promote the prosperity of the siderophore community.

## REFERENCES

1. Francoeur, Paul G., David R. Koes. 2021. “SolTranNet-A Machine Learning Tool for Fast Aqueous Solubility Prediction.” *J Chem Inf Model* 61: 2530-2536. <https://doi.org/10.1021/acs.jcim.1c00331>

2. Evans, Robert, Guilherme Dal Poggetto, Mathias Nilsson, Gareth A. Morris. 2018. “Improving the Interpretation of Small Molecule Diffusion Coefficients.” *Anal Chem* 90: 3987-3994. <https://doi.org/10.1021/acs.analchem.7b05032>

3. Probst, Daniel, Jean-Louis Reymond. 2020. “Visualization of very large high-dimensional data sets as minimum spanning trees.” *Journal of Cheminformatics* 12: <https://doi.org/10.1186/s13321-020-0416-x>

4. Sorokina, Maria, Peter Merseburger, Kohulan Rajan, Mehmet Aziz Yirik, Christoph Steinbeck. 2021. “COCONUT online: Collection of Open Natural Products database.” *Journal of Cheminformatics* 13: 2. <https://doi.org/10.1186/s13321-020-00478-9>

5. Maggiora, Gerald, Martin Vogt, Dagmar Stumpfe, Juergen Bajorath. 2014. “Molecular similarity in medicinal chemistry.” *J Med Chem* 57: 3186-3204. <https://doi.org/10.1021/jm401411z>

6. Schwyn, Bernhard, Joseph B. Neilands. 1987. “Universal chemical assay for the detection and determination of siderophores.” *Anal Biochem* 160: 47-56. <https://doi.org/10.1016/0003-2697(87)90612-9>

7. Hider, Robert C., Xiaole Kong. 2010. “Chemistry and biology of siderophores.” *Natural Product Reports* 27: 637-657. <https://doi.org/10.1039/b906679a>

8. Raines, Daniel J., Olga V. Moroz, Elena V. Blagova, Johan P. Turkenburg, Keith S. Wilson, Anne-K. Duhme-Klair. 2016. “Bacteria in an intense competition for iron: Key component of the <i>Campylobacter jejuni</i> iron uptake system scavenges enterobactin hydrolysis product.” *Proceedings of the National Academy of Sciences* 113: 5850-5855. <https://doi.org/10.1073/pnas.1520829113>

9. Kummerli, Rolf, Konstanze T. Schiessl, Tuija Waldvogel, Kristopher McNeill, Martin Ackermann. 2014. “Habitat structure and the evolution of diffusible siderophores in bacteria.” *Ecology Letters* 17: 1536-1544. <https://doi.org/10.1111/ele.12371>

10. Völker, Christoph, Dieter A. Wolf-Gladrow. 1999. “Physical limits on iron uptake mediated by siderophores or surface reductases.” *Marine Chemistry* 65: 227-244. <https://doi.org/10.1016/S0304-4203(99)00004-3>

11. Demain, Arnold L. 1992. “Microbial Secondary Metabolism - a New Theoretical Frontier for Academia, a New Opportunity for Industry.” *Ciba Foundation Symposia* 171: 3-23. <https://doi.org/10.1002/9780470514344.ch2>

12. Sinsabaugh, Robert L., Brian H. Hill, Jennifer J. Follstad Shah. 2009. “Ecoenzymatic stoichiometry of microbial organic nutrient acquisition in soil and sediment.” *Nature* 462: 795-U117. <https://doi.org/10.1038/nature08632>

13. Chen, Yong-Liang, Lei-Yi Chen, Yun-Feng Peng, Jin-Zhi Ding, Fei Li, Gui-Biao Yang, Dan Kou, et al. 2016. “Linking microbial C:N:P stoichiometry to microbial community and abiotic factors along a 3500-km grassland transect on the Tibetan Plateau.” *Global Ecology and Biogeography* 25: 1416-1427. <https://doi.org/10.1111/geb.12500>

14. Vivanco, Jorge M., Harsh P. Bais, Frank R. Stermitz, Giles C. Thelen, Ragan M. Callaway. 2004. “Biogeographical variation in community response to root allelochemistry: novel weapons and exotic invasion.” *Ecology Letters* 7: 285-292. <https://doi.org/10.1111/j.1461-0248.2004.00576.x>

15. Fazary, Ahmed Eid, Mohamed Taha, Yi-Hsu Ju. 2009. “Iron Complexation Studies of Gallic Acid.” *Journal of Chemical and Engineering Data* 54: 35-42. <https://doi.org/10.1021/je800441u>

16. Matthijs, Sandra, Christine Baysse, Nico Koedam, Kourosch Abbaspour Tehrani, Lauren Verheyden, Herbert Budzikiewicz, Mathias Schafer, et al. 2004. “The Pseudomonas siderophore quinolobactin is synthesized from xanthurenic acid, an intermediate of the kynurenine pathway.” *Molecular Microbiology* 52: 371-384. <https://doi.org/10.1111/j.1365-2958.2004.03999.x>

17. Barraza, Monique L., Carl E. Coppock, Kelly N. Brooks, Donna L. Wilks, Richard G. Saunders, George W. Latimer. 1991. “Iron Sulfate and Feed Pelleting to Detoxify Free Gossypol in Cottonseed Diets for Dairy-Cattle.” *Journal of Dairy Science* 74: 3457-3467. <https://doi.org/10.3168/jds.S0022-0302(91)78536-6>

18. Mossialos, Dimitris, Jean-Marie Meyer, Herbert Budzikiewicz, Ulrich Wolff, Nico Koedam, Christine Baysse, Vanamala Anjaiah, Pierre Cornelis. 2000. “Quinolobactin, a New Siderophore of<i>Pseudomonas fluorescens</i> ATCC 17400, the Production of Which Is Repressed by the Cognate Pyoverdine.” *Applied and Environmental Microbiology* 66: 487-492. <https://doi.org/10.1128/AEM.66.2.487-492.2000>

19. Matthijs, Sandra, Kourosch Abbaspour Tehrani, George Laus, Robert W. Jackson, Richard M. Cooper, Pierre Cornelis. 2007. “Thioquinolobactin, a Pseudomonas siderophore with antifungal and anti-Pythium activity.” *Environmental Microbiology* 9: 425-434. <https://doi.org/10.1111/j.1462-2920.2006.01154.x>

20. 石井, 永, 益昇 陳, 美砂子 赤池, 勉 石川, 盛徳 盧. 1982. “ミカン科植物成分の研究(第44報)台湾産ネワタノキXanthoxylum integrifoliolum(MERR.)MERR.(Fagara integrifoliola MERR.)の成分検索 その1 根木質部の成分.” *藥學雜誌* 102: 182-195. <https://doi.org/10.1248/yakushi1947.102.2_182>

21. Chen, Jih-Jung, Tzu-Ying Wang, Tsong-Long Hwang. 2008. “Neolignans, a Coumarinolignan, Lignan Derivatives, and a Chromene: Anti-inflammatory Constituents from Zanthoxylum avicennae.” *Journal of Natural Products* 71: 212-217. <https://doi.org/10.1021/np070594k>

22. Moon, Surk-Sik, Namsun Cho, Jongheon Shin, Youngwan Seo, Chong Ock Lee, Sang Un Choi. 1996. “Jineol, a Cytotoxic Alkaloid from the Centipede Scolopendra subspinipes.” *Journal of Natural Products* 59: 777-779. <https://doi.org/10.1021/np960188t>

23. Kramer, Jos, Özhan Özkaya, Rolf Kümmerli. 2020. “Bacterial siderophores in community and host interactions.” *Nature Reviews Microbiology* 18: 152–163. <https://doi.org/10.1038/s41579-019-0284-4>

24. Cordero, Otto X., Laure-Anne Ventouras, Edward F. DeLong, Martin F. Polz. 2012. “Public good dynamics drive evolution of iron acquisition strategies in natural bacterioplankton populations.” *Proceedings of the National Academy of Sciences of the United States of America* 109: 20059-20064. <https://doi.org/10.1073/pnas.1213344109>

25. Butaite, Elena, Michael Baumgartner, Stefan Wyder, Rolf Kummerli. 2017. “Siderophore cheating and cheating resistance shape competition for iron in soil and freshwater Pseudomonas communities.” *Nature Communications* 8: <https://doi.org/10.1038/s41467-017-00509-4>

26. Ozkaya, Ozhan, Roberto Balbontin, Isabel Gordo, Karina B. Xavier. 2018. “Cheating on Cheaters Stabilizes Cooperation in Pseudomonas aeruginosa.” *Current Biology* 28: 2070-+. <https://doi.org/10.1016/j.cub.2018.04.093>

27. Shepherdson, Evan M. F., Marie A. Elliot. 2022. “Cryptic specialized metabolites drive <i>Streptomyces</i> exploration and provide a competitive advantage during growth with other microbes.” *Proceedings of the National Academy of Sciences* 119: e2211052119. <https://doi.org/doi:10.1073/pnas.2211052119>

28. D'Onofrio, Anthony, Jason M. Crawford, Eric J. Stewart, Kathrin Witt, Ekaterina Gavrish, Slava Epstein, Jon Clardy, Kim Lewis. 2010. “Siderophores from Neighboring Organisms Promote the Growth of Uncultured Bacteria.” *Chemistry & Biology* 17: 254-264. <https://doi.org/10.1016/j.chembiol.2010.02.010>

29. Maglangit, Fleurdeliz, Ming Him Tong, Marcel Jaspars, Kwaku Kyeremeh, Hai Deng. 2019. “Legonoxamines A-B, two new hydroxamate siderophores from the soil bacterium, Streptomyces sp. MA37.” *Tetrahedron Letters* 60: 75-79. <https://doi.org/10.1016/j.tetlet.2018.11.063>

30. Wilson, Melissa K., Rebecca J. Abergel, Kenneth N. Raymond, Jean E. L. Arceneaux, B. Rowe Byers. 2006. “Siderophores of Bacillus anthracis, Bacillus cereus, and Bacillus thuringiensis.” *Biochemical and Biophysical Research Communications* 348: 320-325. <https://doi.org/10.1016/j.bbrc.2006.07.055>

31. Zajdowicz, Sheryl, Jon C. Haller, Amy E. Krafft, Steve W. Hunsucker, Colin T. Mant, Mark W. Duncan, Robert S. Hodges, David N. M. Jones, Randall K. Holmes. 2012. “Purification and Structural Characterization of Siderophore (Corynebactin) from Corynebacterium diphtheriae.” *Plos One* 7: e34591. <https://doi.org/10.1371/journal.pone.0034591>

32. Patzer, Silke I., Volkmar Braun. 2010. “Gene Cluster Involved in the Biosynthesis of Griseobactin, a Catechol-Peptide Siderophore of Streptomyces sp ATCC 700974.” *Journal of Bacteriology* 192: 426-435. <https://doi.org/10.1128/Jb.01250-09>

33. Matsuo, Yoshihide, Kaneo Kanoh, Jae-Hyuk Jang, Kyoko Adachi, Satoru Matsuda, Osamu Miki, Toshiaki Kato, Yoshikazu Shizuri. 2011. “Streptobactin, a Tricatechol-Type Siderophore from Marine-Derived Streptomyces sp YM5-799.” *Journal of Natural Products* 74: 2371-2376. <https://doi.org/10.1021/np200290j>

34. Carrero, Manuel, Felix J. Sangari, Jesus Aguero, Juan M Garcı́a Lobo. 2002. “Brucella abortus strain 2308 produces brucebactin, a highly efficient catecholic siderophore.” *Microbiology-Sgm* 148: 353-360. <https://doi.org/10.1099/00221287-148-2-353>

35. Miller, Amanda Lynn, Shanren Li, Catherine D. Eichhorn, Yongbiao Zheng, Liangcheng Du. 2023. “Identification and Biosynthetic Study of the Siderophore Lysochelin in the Biocontrol Agent Lysobacter enzymogenes.” *Journal of Agricultural and Food Chemistry* <https://doi.org/10.1021/acs.jafc.3c01250>

36. Zheng, Shuangjia, Tao Zeng, Chengtao Li, Binghong Chen, Connor W. Coley, Yuedong Yang, Ruibo Wu. 2022. “Deep learning driven biosynthetic pathways navigation for natural products with BioNavi-NP.” *Nature Communications* 13: <https://doi.org/10.1038/s41467-022-30970-9>

37. Isaka, Masahiko, Morakot Tanticharoen, Puapong Kongsaeree, Yodhathai Thebtaranonth. 2001. “Structures of cordypyridones A-D, antimalarial N-hydroxy- and N-methoxy-2-pyridones from the insect pathogenic fungus Cordyceps nipponica.” *Journal of Organic Chemistry* 66: 4803-4808. <https://doi.org/10.1021/jo0100906>

38. Pita-Grisanti, Valentina, Kaylin Chasser, Trevor Sobol, Zobeida Cruz-Monserrate. 2022. “Understanding the Potential and Risk of Bacterial Siderophores in Cancer.” *Frontiers in Oncology* 12: <https://doi.org/10.3389/fonc.2022.867271>

39. Ganz, Tomas, Elizabeta Nemeth. 2015. “Iron homeostasis in host defence and inflammation.” *Nature Reviews Immunology* 15: 500-510. <https://doi.org/10.1038/nri3863>

40. Nairz, Manfred, Guenter Weiss. 2020. “Iron in infection and immunity.” *Molecular Aspects of Medicine* 75: <https://doi.org/10.1016/j.mam.2020.100864>

41. Chuljerm, Hataichanok, Manu Deeudom, Suthat Fucharoen, Francesca Mazzacuva, Robert C. Hider, Somdet Srichairatanakool, Agostino Cilibrizzi. 2020. “Characterization of two siderophores produced by Bacillus megaterium: A preliminary investigation into their potential as therapeutic agents.” *Biochimica Et Biophysica Acta-General Subjects* 1864: <https://doi.org/10.1016/j.bbagen.2020.129670>

42. Achard, Maud E. S., Kaiwen W. Chen, Matthew J. Sweet, Rebecca E. Watts, Kate Schroder, Mark A. Schembri, Alastair G. McEwan. 2013. “An antioxidant role for catecholate siderophores in Salmonella.” *Biochemical Journal* 454: 543-549. <https://doi.org/10.1042/Bj20121771>

43. Peralta, Daiana R., Conrado Adler, Natalia S. Corbalan, Enrique Carlos Paz Garcia, Maria Fernanda Pomares, Paula A. Vincent. 2016. “Enterobactin as Part of the Oxidative Stress Response Repertoire.” *Plos One* 11: <https://doi.org/10.1371/journal.pone.0157799>

44. Hesse, Elze, Siobhan O'Brien, Nicolas Tromas, Florian Bayer, Adela M. Lujan, Eleanor M. van Veen, Dave J. Hodgson, Angus Buckling. 2018. “Ecological selection of siderophore-producing microbial taxa in response to heavy metal contamination.” *Ecology Letters* 21: 117-127. <https://doi.org/10.1111/ele.12878>

45. Roskova, Zuzana, Radek Skarohlid, Lenka McGachy. 2022. “Siderophores: an alternative bioremediation strategy?” *Sci Total Environ* 819: 153144. <https://doi.org/10.1016/j.scitotenv.2022.153144>

### Supplementary figures


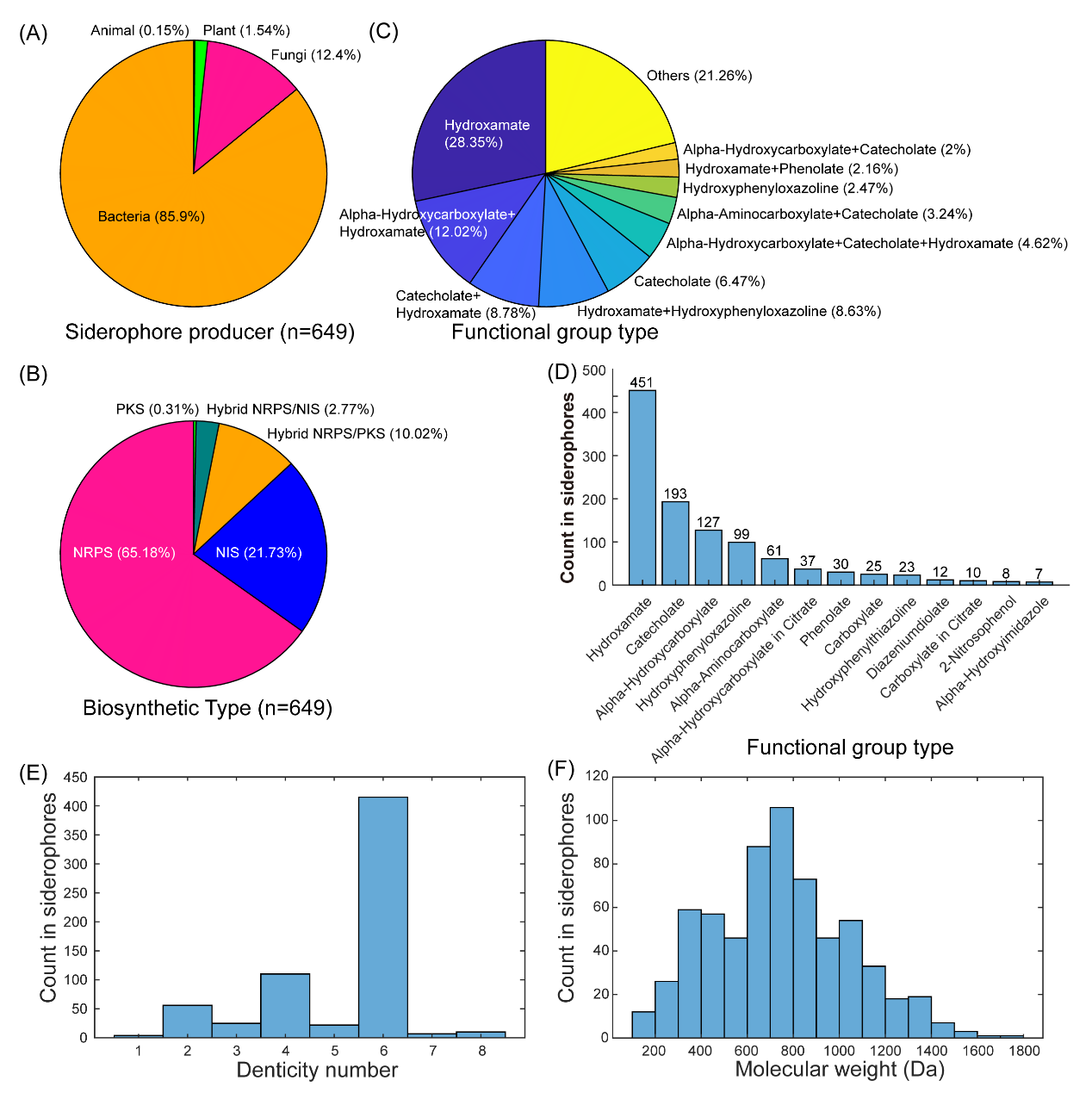


**Figure S1 The statistics of 649 unique siderophores in SIDERITE**. (A) Distribution of the siderophore producers by their kingdoms. (B) Distribution of the siderophore biosynthetic pathways. (C) Distribution of the functional group type combinations. For clarity, only the top ten combinations are shown, and the others are merged into "Others". (D) Distribution of the common functional group of siderophores. One siderophore could contribute to more than one functional group type if it contains many types of functional groups. (E) Distribution of denticity numbers. (F) Distribution of the molecular weight.


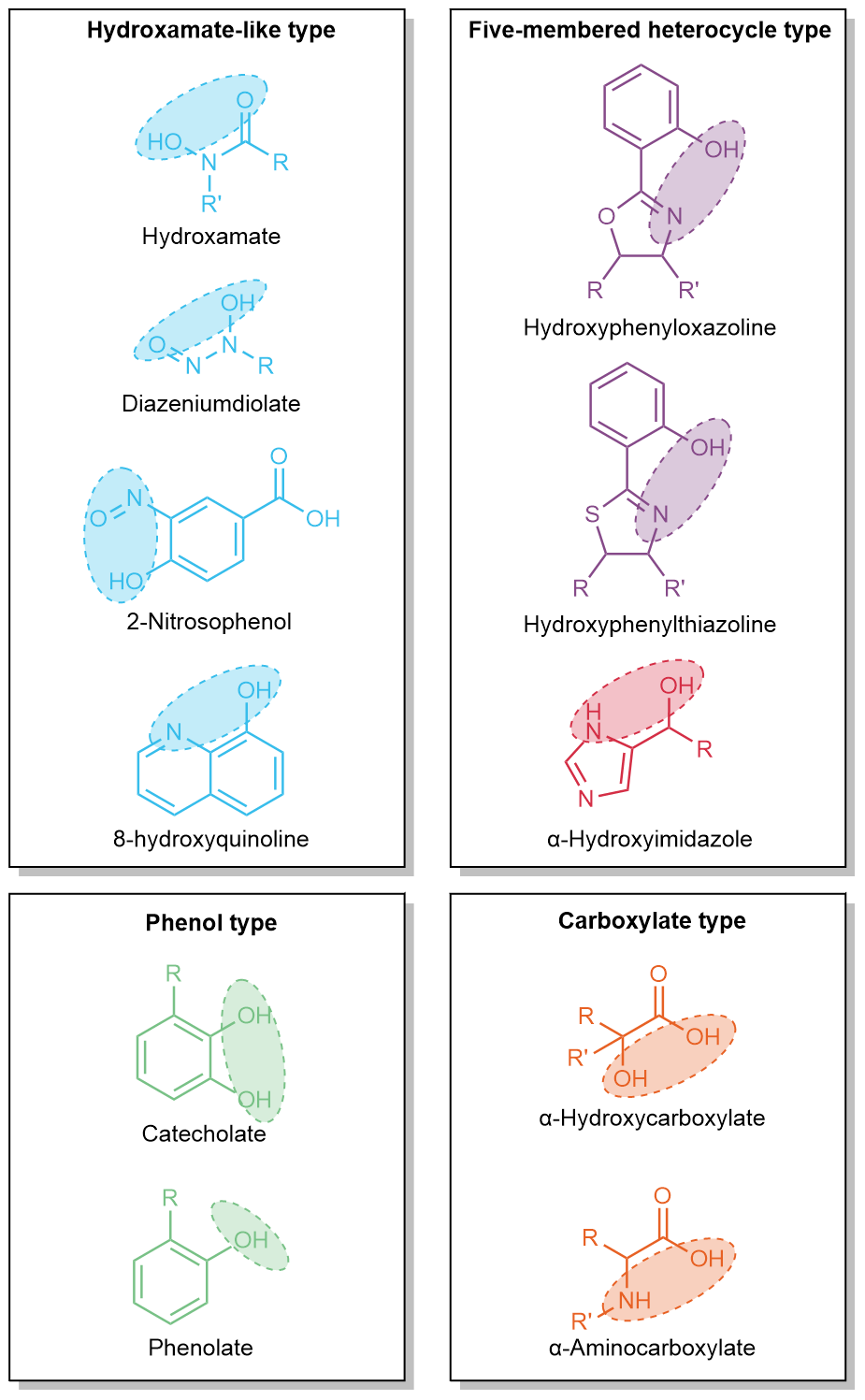


**Figure S2 Known siderophore functional groups (ligands).**


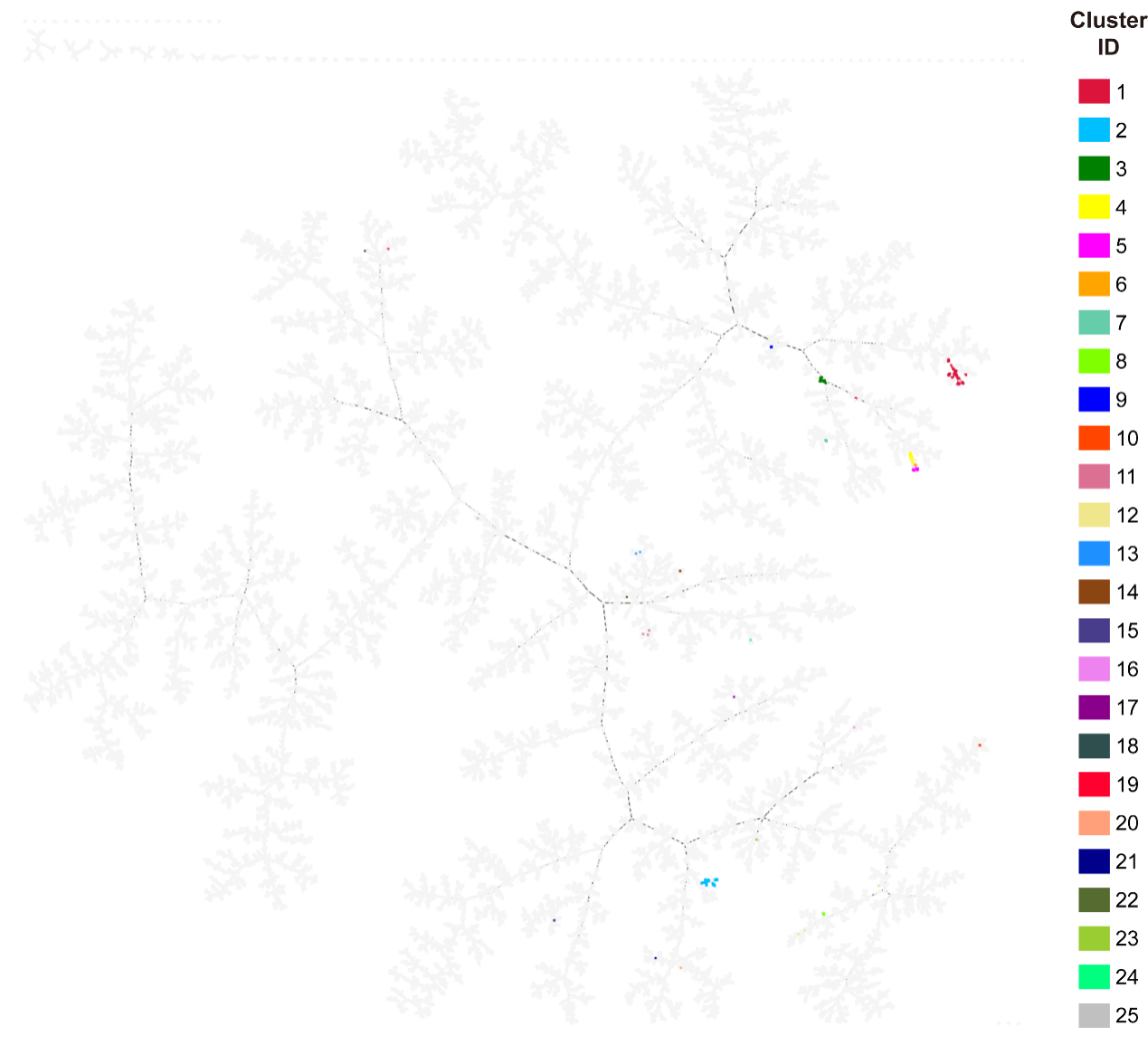


**Figure S3 Displaying 25 clusters of 649 siderophores in the COCONUT database by TMAP.**


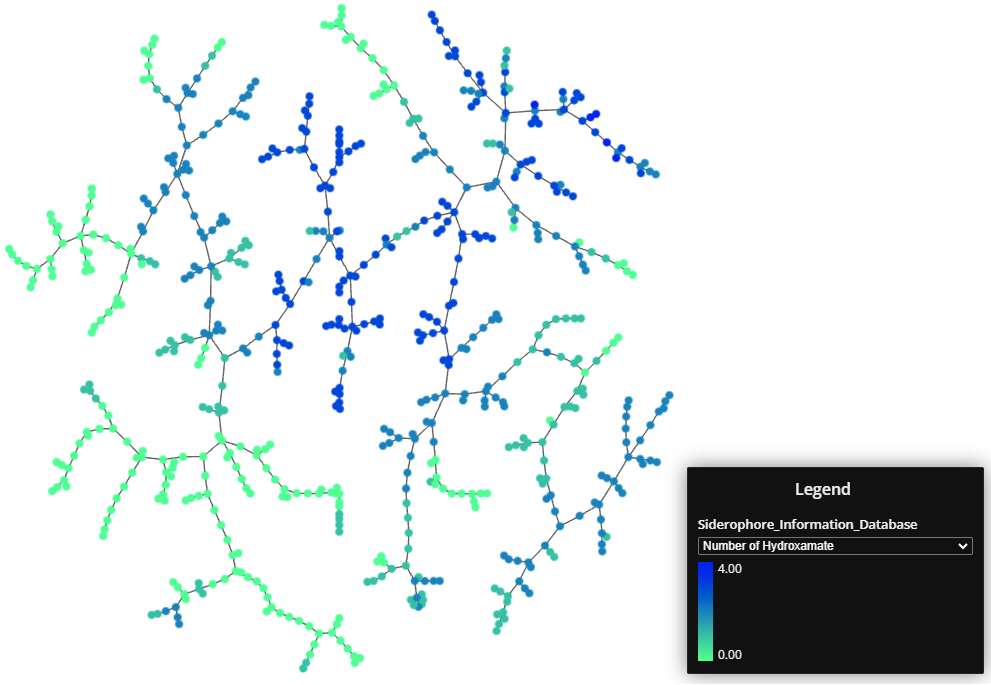


**Figure S4 Visualization of 649 siderophores with functional group hydroxamate number by TAMP.**


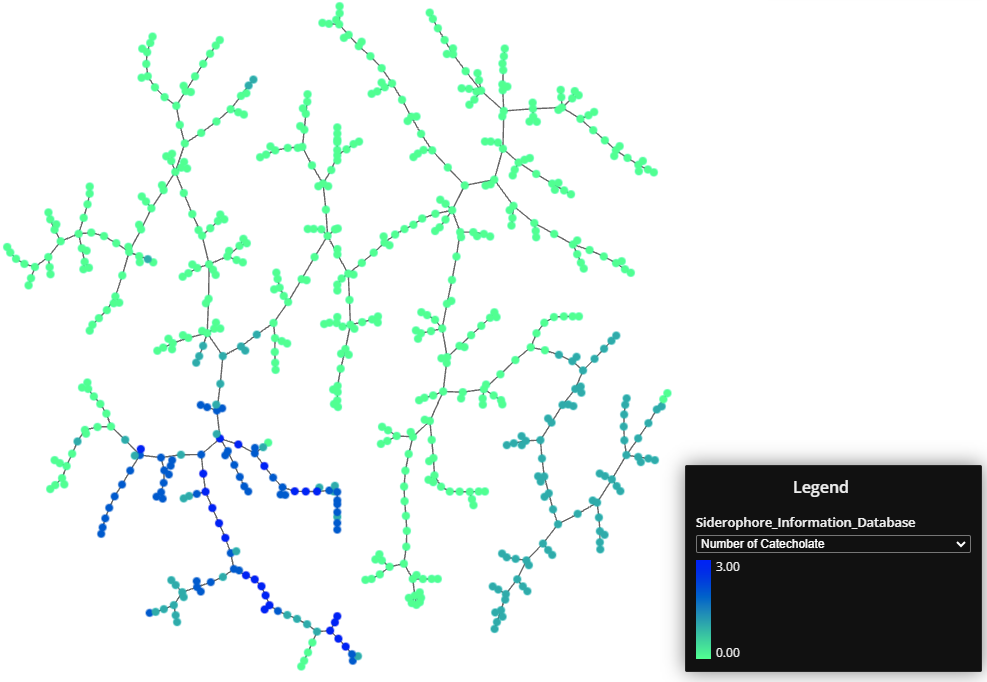


**Figure S5 Visualization of 649 siderophores with functional group catecholate number by TAMP.**


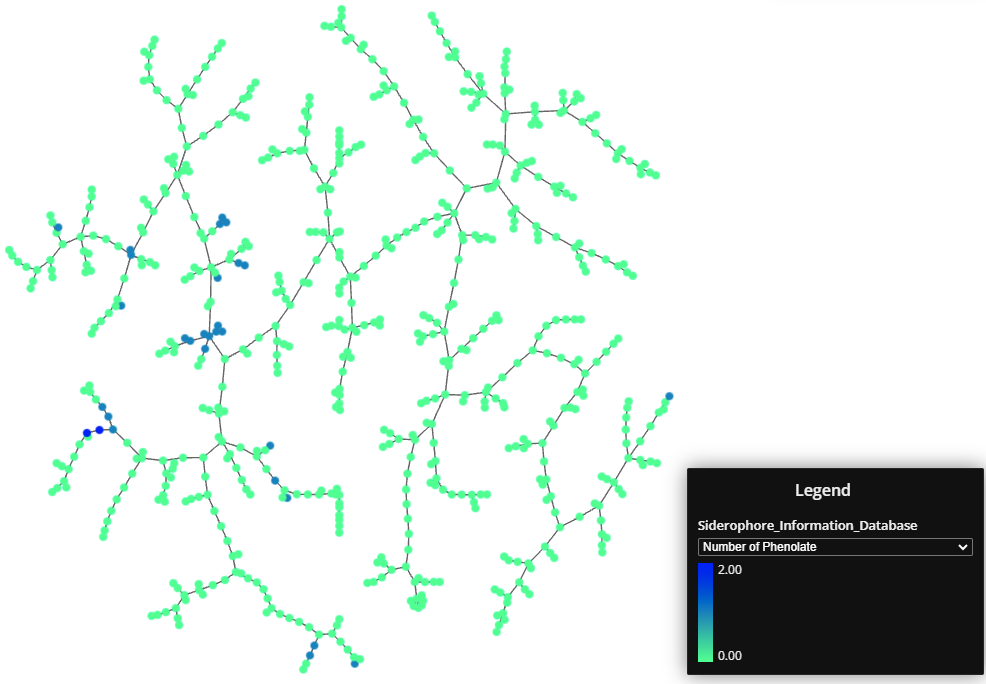


**Figure S6 Visualization of 649 siderophores with functional group phenolate number by TAMP.**


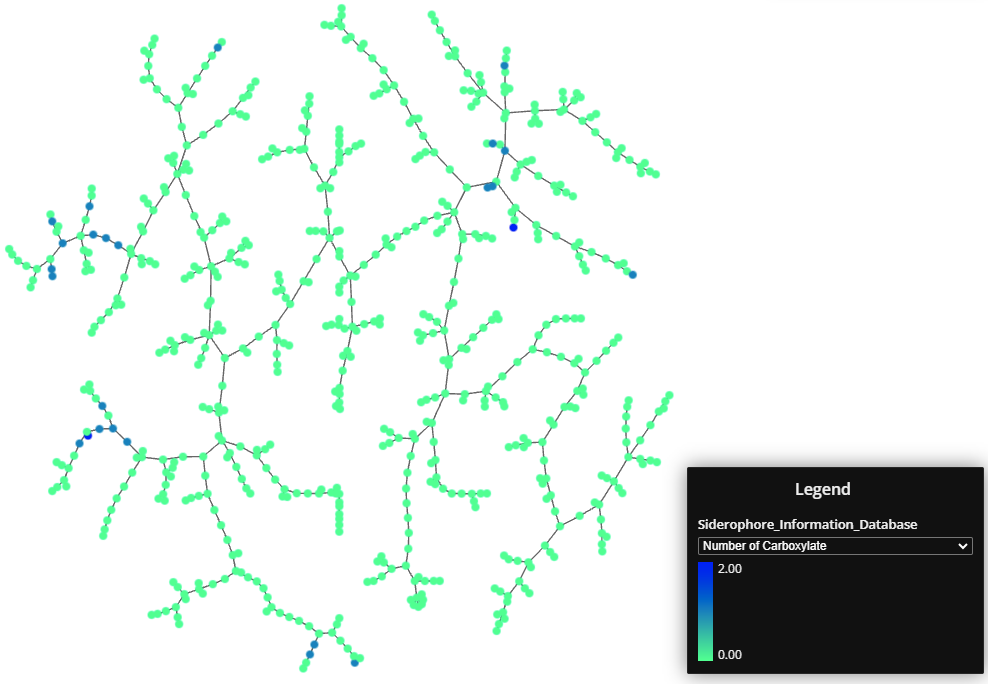


**Figure S7 Visualization of 649 siderophores with functional group carboxylate number by TAMP.**


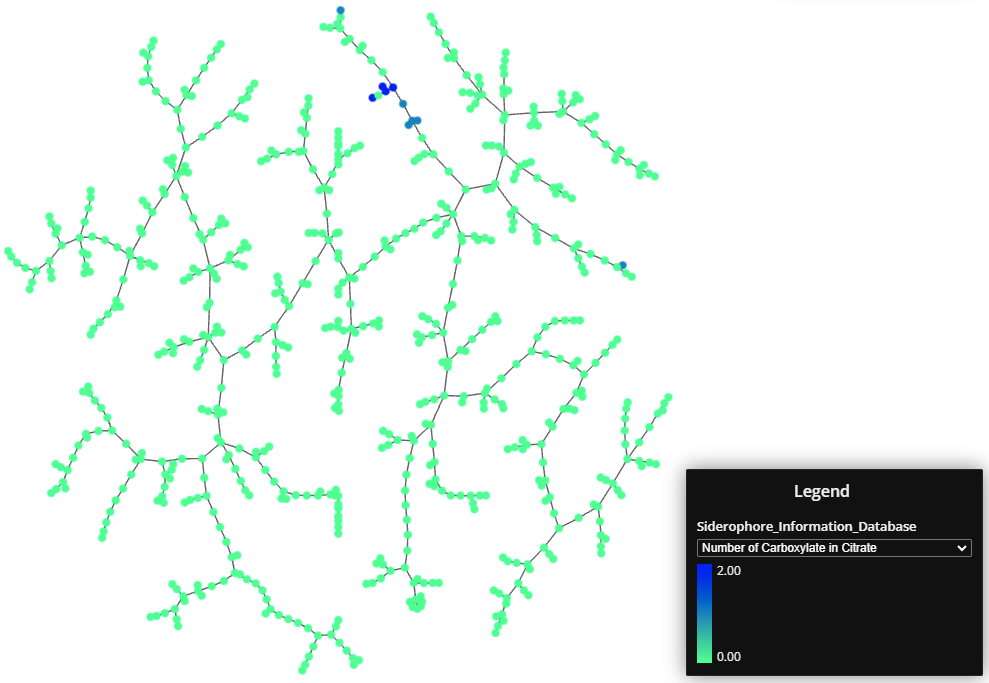


**Figure S8 Visualization of 649 siderophores with functional group carboxylate in citrate number by TAMP.**


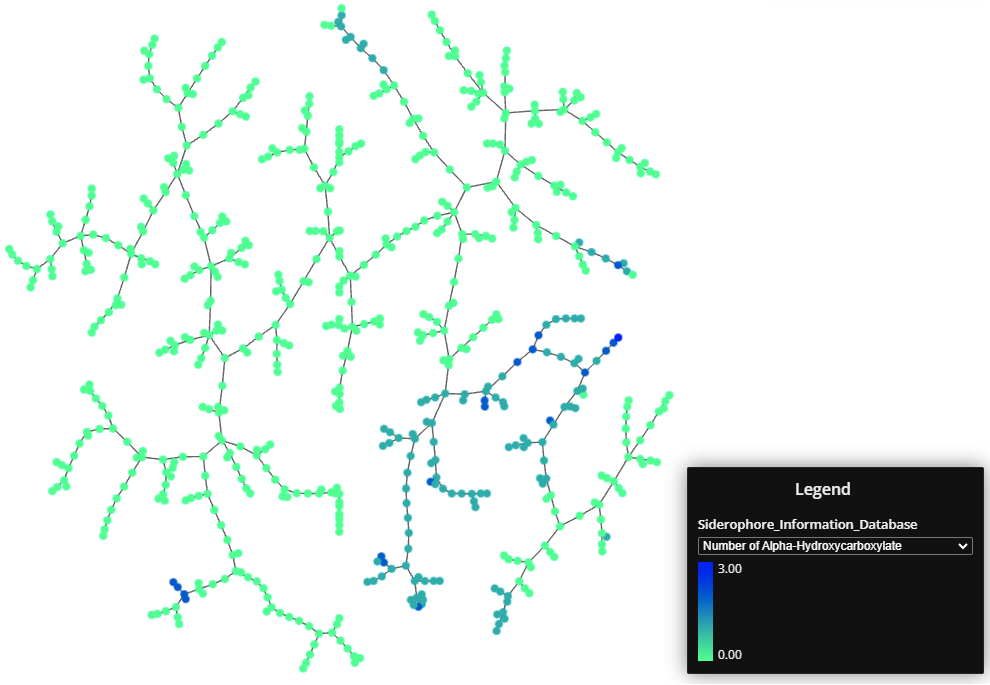


**Figure S9 Visualization of 649 siderophores with functional group alpha-hydroxycarboxylate number by TAMP.**


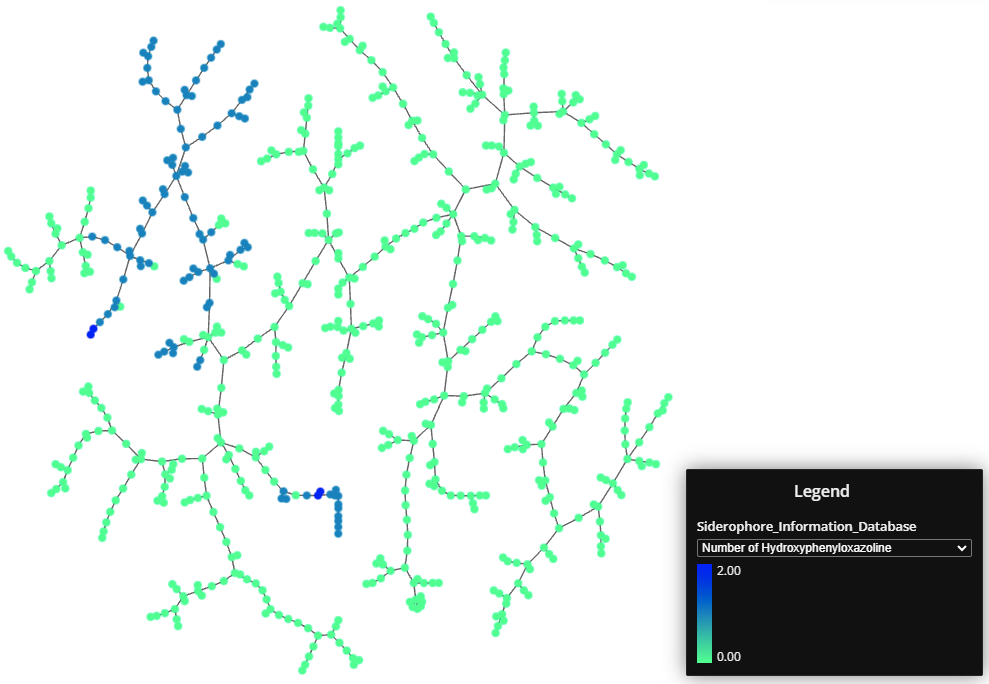


**Figure S10 Visualization of 649 siderophores with functional group hydroxyphenyloxazoline number by TAMP.**


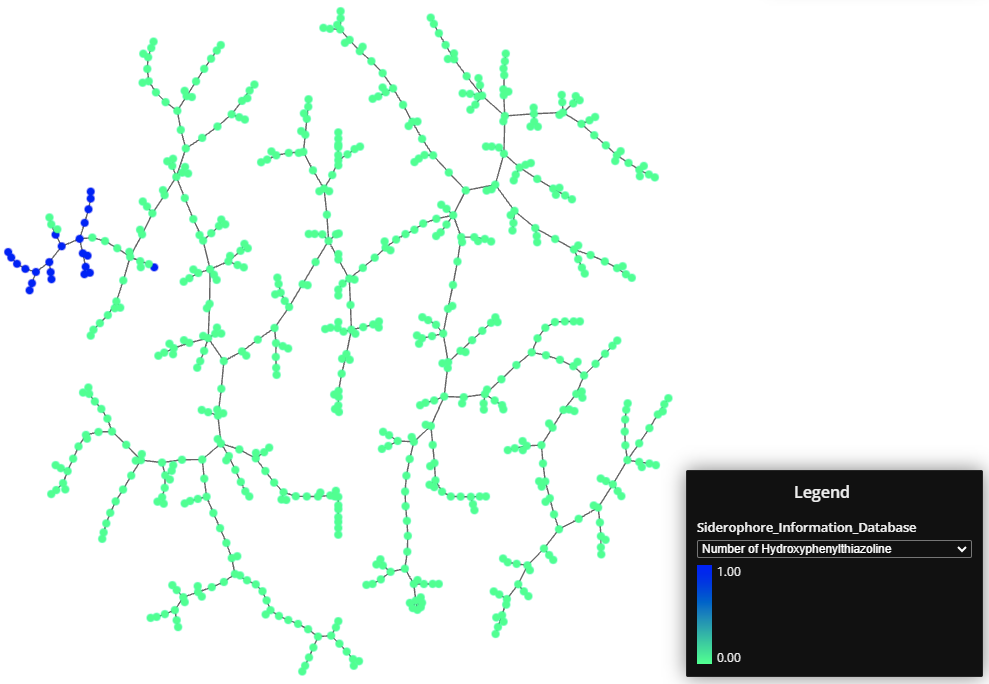


**Figure S11 Visualization of 649 siderophores with functional group hydroxyphenylthiazoline number by TAMP.**


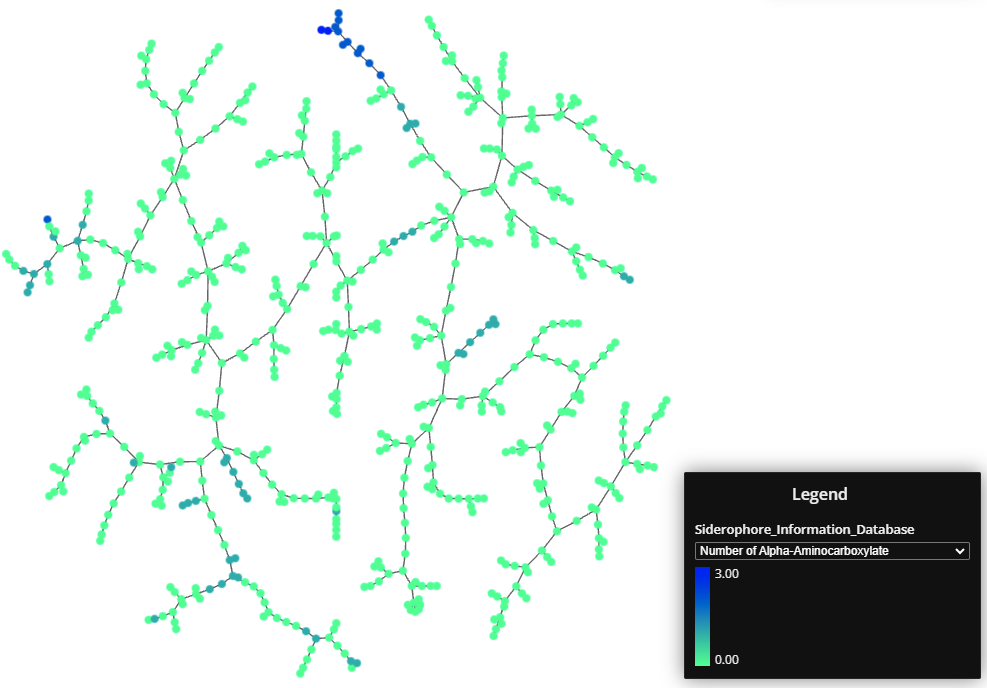


**Figure S12 Visualization of 649 siderophores with functional group alpha-aminocarboxylate number by TAMP.**


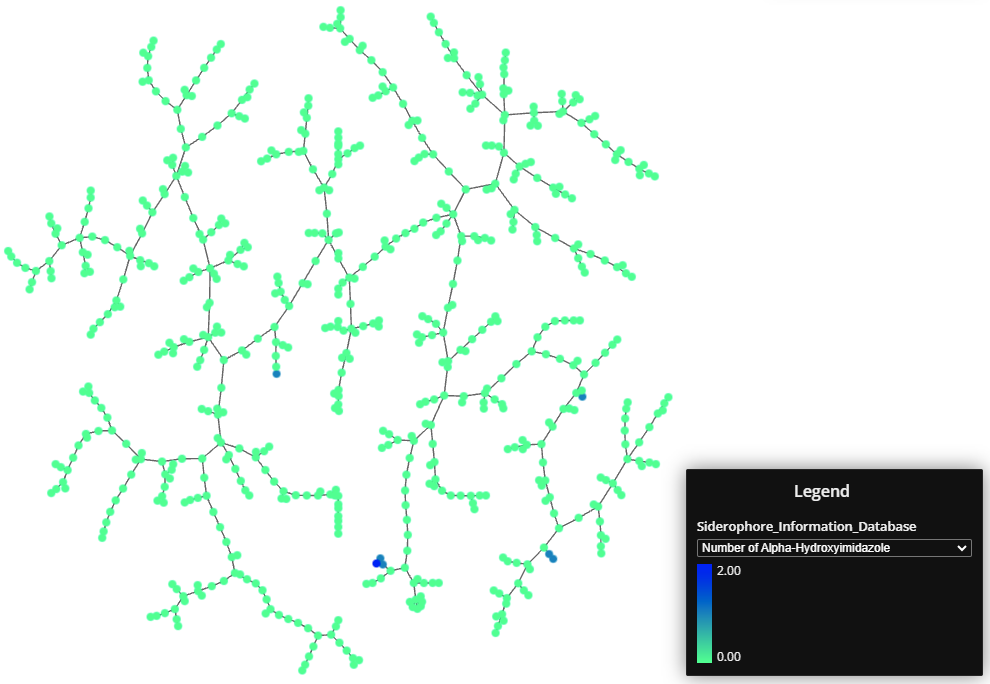


**Figure S13 Visualization of 649 siderophores with functional group alpha-hydroxyimidazole number by TAMP.**


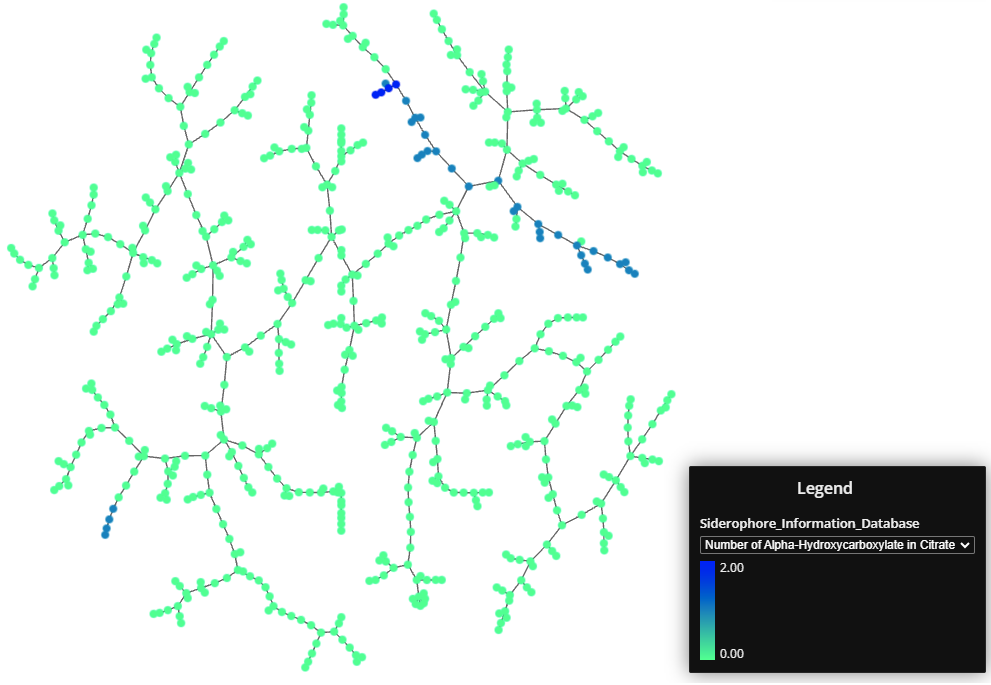


**Figure S14 Visualization of 649 siderophores with functional group alpha-hydroxycarboxylate in citrate number by TAMP.**


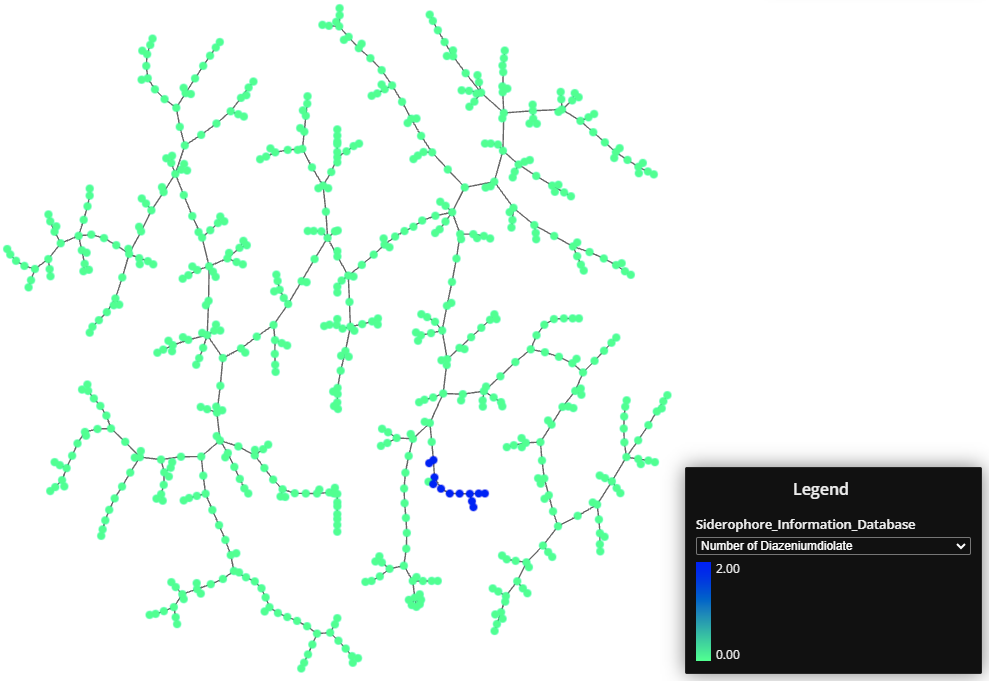


**Figure S15 Visualization of 649 siderophores with functional group diazeniumdiolate number by TAMP.**


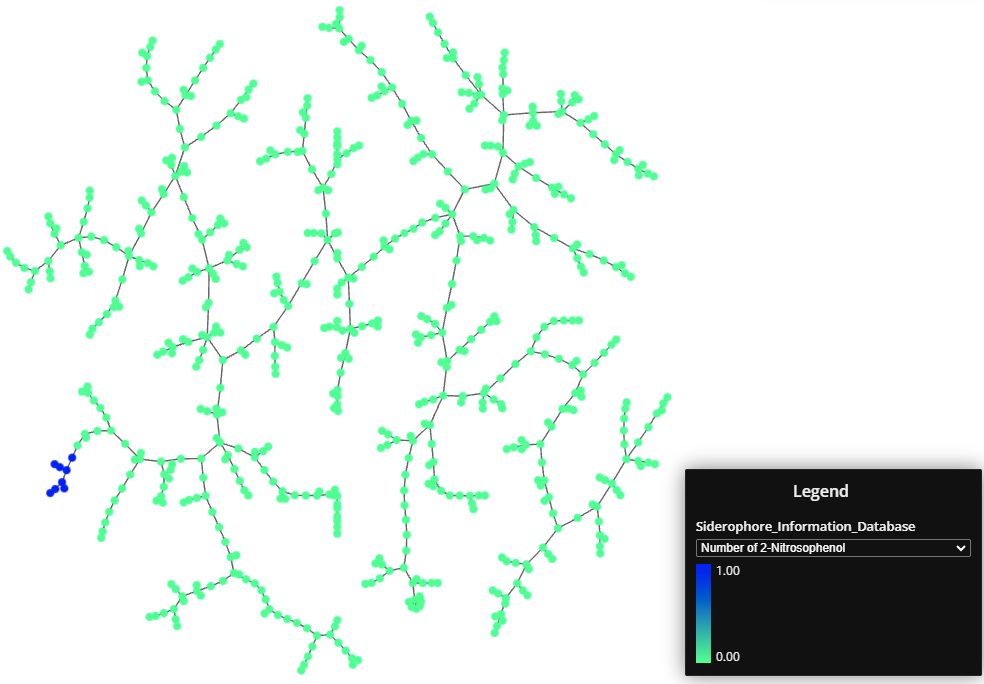


**Figure S16 Visualization of 649 siderophores with functional group 2-nitrosophenol number by TAMP.**


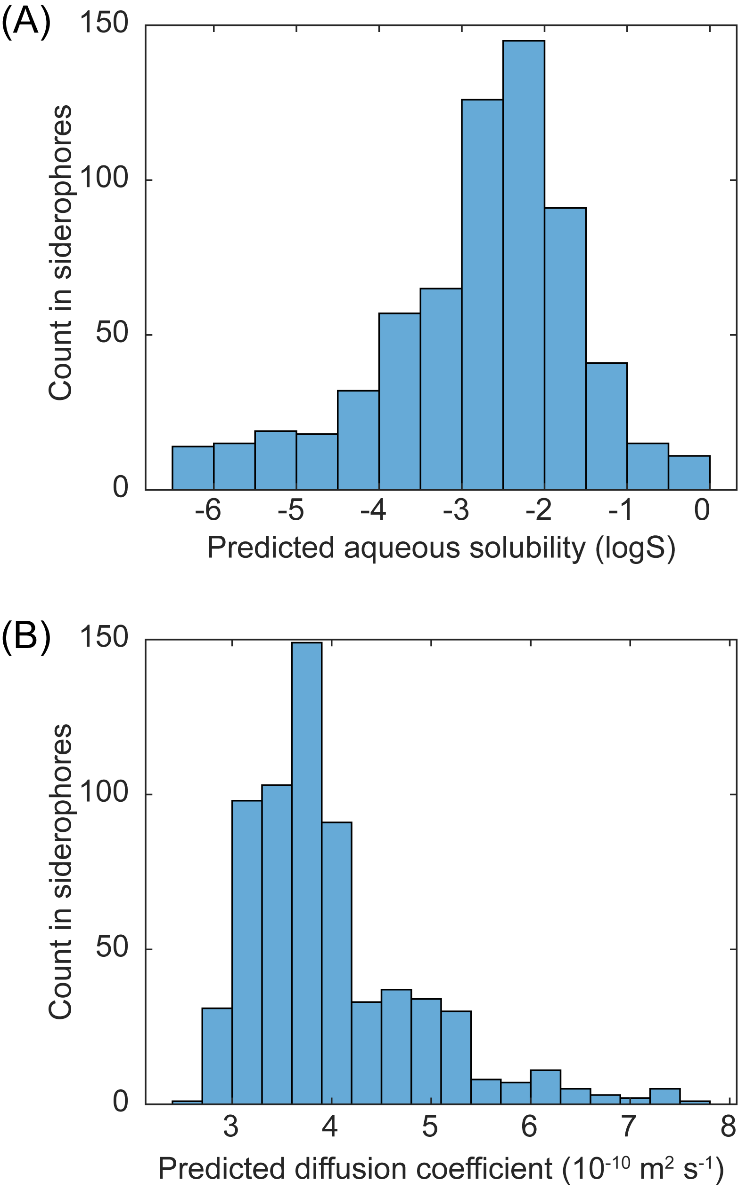


**Figure S17 The predicted properties of 649 siderophores**. (A) The predicted aqueous solubility. The unit is log10 of solubility in the water. (B) The predicted diffusion coefficient. The temperature is set to 298.15K and the solvent is water.


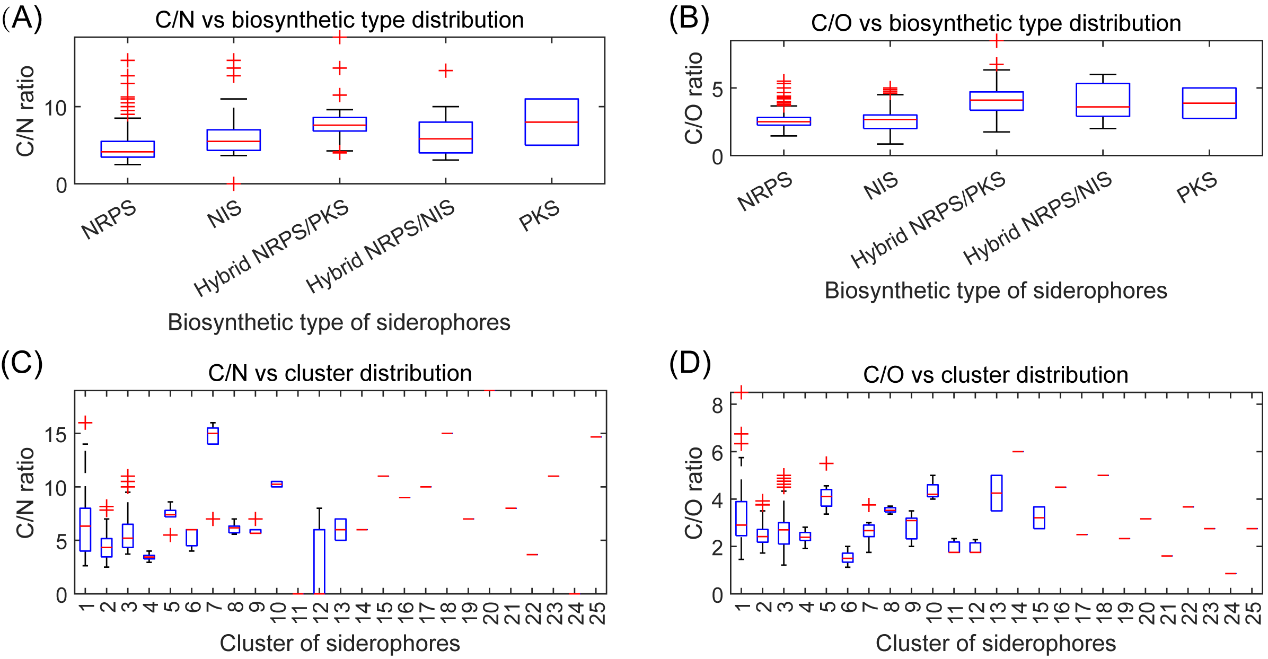


**Figure S18 The distribution of C/N and C/O ratios in the different biosynthetic types and clusters**. (A) The distribution of C/N ratio between different biosynthetic types of siderophores. (B) The distribution of C/O ratio between different biosynthetic types of siderophores. (C) The distribution of C/N ratio between different clusters of siderophores. (D) The distribution of C/O ratio between different clusters of siderophores.


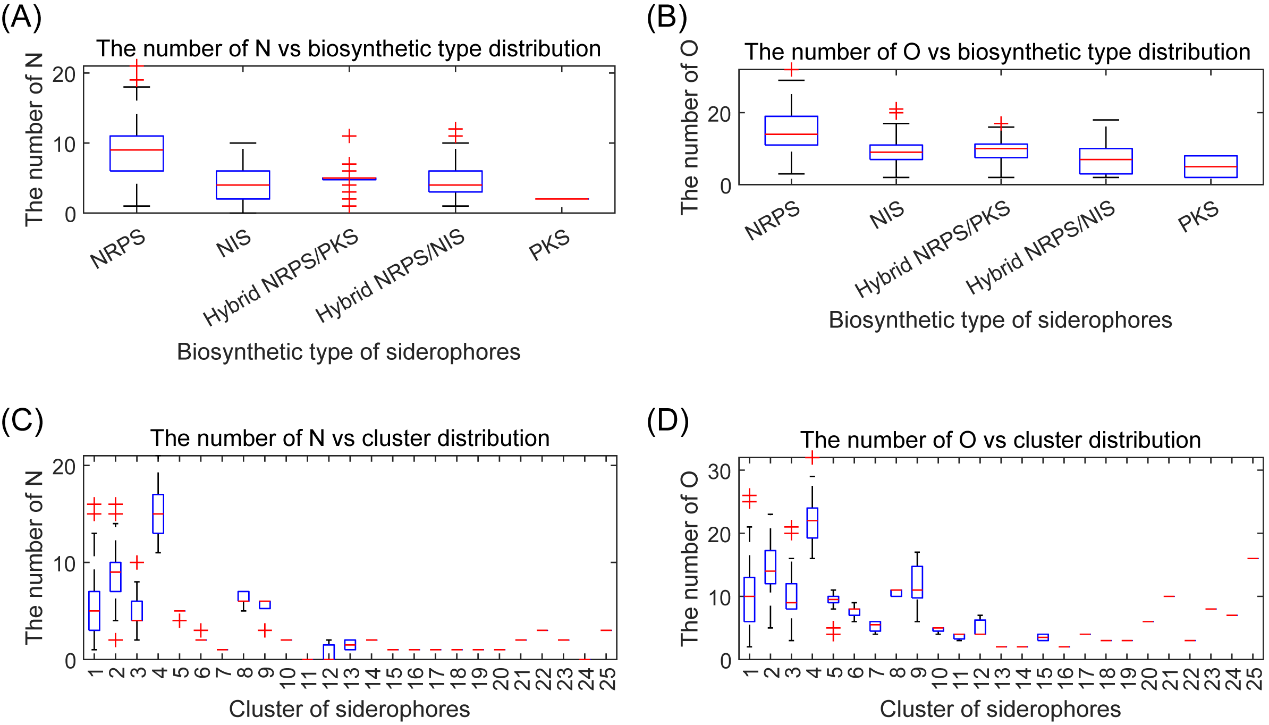


**Figure S19 The distribution of nitrogen atom and oxygen atom numbers in the different biosynthetic types and clusters**. (A) The distribution of nitrogen atom number between different biosynthetic types of siderophores. (B) The distribution of oxygen atom number between different biosynthetic types of siderophores. (C) The distribution of nitrogen atom number between different clusters of siderophores. (D) The distribution of oxygen atom number between different clusters of siderophores.


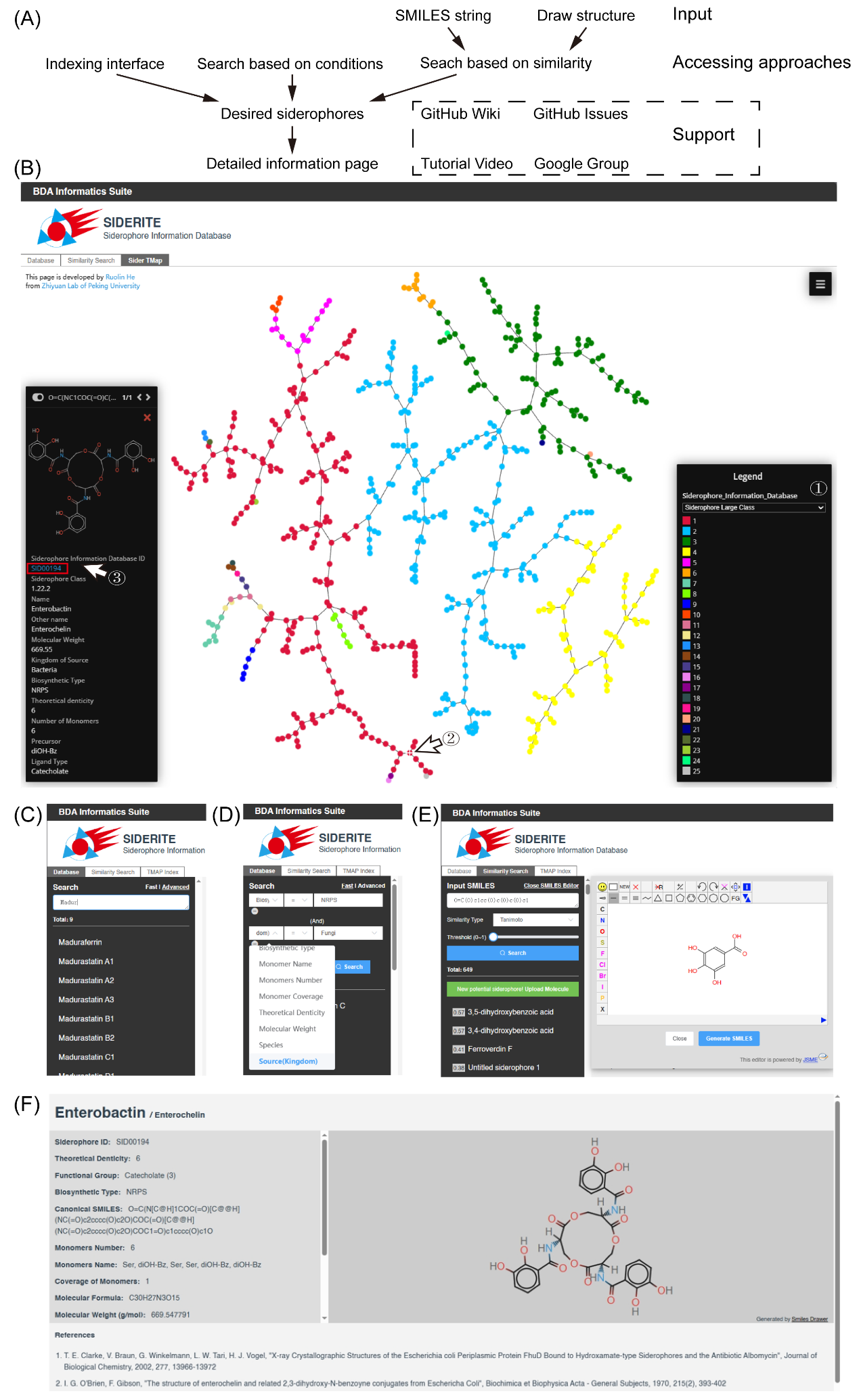


**Figure S20 The SIDERITE database usage and interface**. (A) Workflow of SIDERITE database usage. (B) Accessing siderophores by the indexing interface by the following steps: 1. Choose a type of siderophore legend. 2. Click the interested siderophore. 3. Jump to the siderophore page by the interactive siderophore ID in the information card. (C) Fast fuzzy siderophore search. (D) Advanced siderophore search by precise conditions. (E) Chemical similarity search, where users can type the SMILES string or draw in the SMILES editor. (F) The individual page with detailed information on each siderophore structure.
